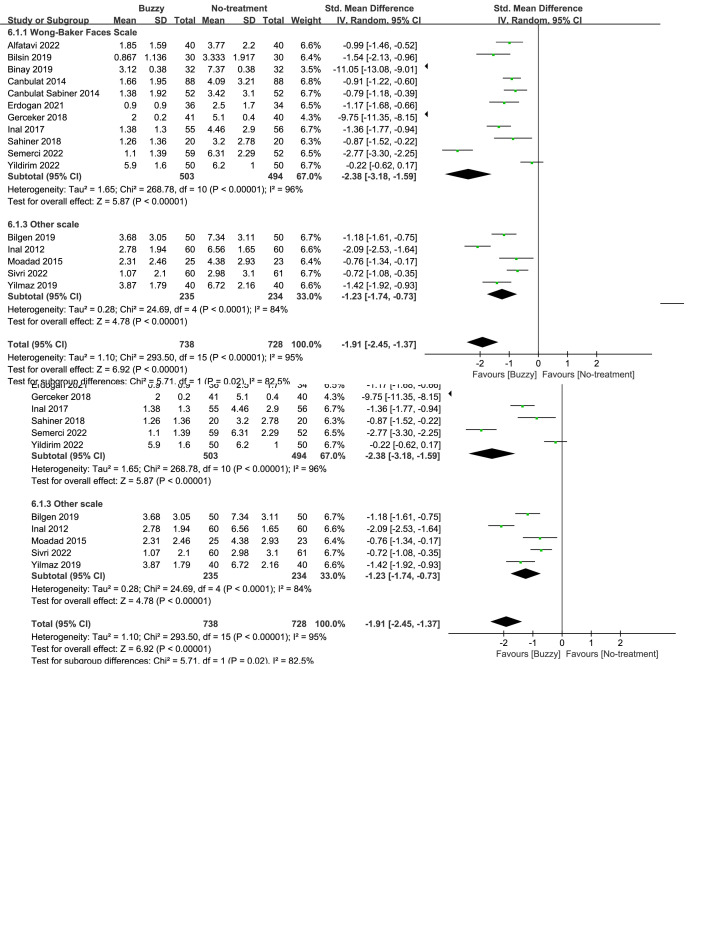# Effectiveness and safety of Buzzy device in needle-related procedures for children under twelve years of age: A systematic review and meta-analysis

**DOI:** 10.1097/MD.0000000000037522

**Published:** 2024-04-12

**Authors:** Faguang Jin, Xiaofang Wang, Maomao Qi, Wenhua Zhang, Yongfeng Zhang

**Affiliations:** a Clinical Medical College, Weifang Medical University, Weifang, China; b Department of Pediatrics, Affiliated Hospital of Weifang Medical University, Weifang, China.

**Keywords:** buzzy, children, needle-related procedures

## Abstract

**Background:**

Pain transcends simple physiology, encompassing biological, emotional, psychological, and social facets. Children show pronounced immediate and enduring responses to pain-related procedures. The aim of this meta-analysis is to investigate the efficacy and safety of the Buzzy device for needle-related procedures in children aged twelve years or younger.

**Methods:**

PubMed, Web of Science, and Embase were searched from inception to July 2023. Only randomized controlled trials utilizing the Buzzy device for needle-related procedures in children under twelve years old were included. Two reviewers independently conducted study selection, data extraction, and risk of bias assessment. Random-effects models were utilized, and analyses were performed using mean differences or standardized mean differences as well as risk ratios.

**Results:**

A total of 19 studies were included, involving 2846 participants (Buzzy = 1095, Control = 1751). Compared to no intervention, the Buzzy device significantly reduced pain response [self-report SMD = −1.90 (−2.45, −1.36), parental SMD = −3.04 (−4.09, −1.99), observer SMD = −2.88 (−3.75, −2.02)] and anxiety scores [self-report SMD = −1.97 (−3.05, −0.88), parental SMD = −2.01 (−2.93, −1.08), observer SMD = −1.92 (−2.64, −1.19)]. Compared to virtual reality (VR), the Buzzy device reduced self-reported anxiety levels SMD = −0.47 (−0.77, −0.17), and compared to distraction cards, the Buzzy device reduced parental and observer-reported pain [parental SMD = −0.85 (−1.22, −0.48), observer SMD = −0.70 (−1.00, −0.40)] and anxiety [parental SMD = −0.96 (−1.46, −0.47), observer SMD = −0.91 (−1.40, −0.42)]. Subgroup analysis results showed that procedure type, patient age, measurement scales used, and distance of operation were not the reason of heterogeneity. The summarized first puncture attempt success rate did not differ from other interventions. There were no significant adverse events in the included studies.

**Conclusion:**

The Buzzy device reduces pain and anxiety in children during needle procedures, ensuring success and safety. Additionally, the effectiveness of the Buzzy device in reducing pain during venipuncture is superior when compared to its effectiveness during intramuscular injections.

## 1. Introduction

Needle procedures are essential in pediatric medical care. According to the immunization schedule provided by the Centers for Disease Control and Prevention, children adhering to medical recommendations are set to receive over 20 vaccinations from birth to 15 months.^[1]^ Hospitalized children frequently require additional needle-related procedures like blood sampling and infusion treatments due to their medical conditions. Although these procedures are necessary, they often prove to be distressing experiences for children with developing cognitive abilities.^[2]^ Feedback from caregivers indicates that of the children aged 6 to 17 with type 1 diabetes undergoing multiple daily injections, a substantial 32.7% manifest needle aversion. Moreover, 22.2% of teenagers aged 11 to 17 express significant apprehension towards needles.^[3]^ Needle procedures during childhood can have profound implications for both their physical and mental well-being.^[4]^ In the short term, needle-related interventions lead to heightened pain perception and discomfort during the procedure, intensifying physical distress and psychological burden. Such experiences can lead to emotional instability characterized by anxiety, fear, and, in some cases, acute emotional distress, which can disrupt emotional well-being and psychological balance.^[5]^ Additionally, needle-associated interventions can potentially trigger enduring psychological trauma including mood disorders, social disorders, and even affects quality of life and social interactions.

Various pharmacological and non-pharmacological interventions have been studied to manage needle-related pain in pediatric settings.^[5]^ However, debate continues about the safety and effectiveness of these interventions. Common pharmacological approaches encompass the use of topical anesthetic patches (e.g. Eutectic Mixture of Local Anesthetics [EMLA]) and cooling sprays. Non-pharmacological strategies include distraction cards, animated media, virtual reality (VR), and ShotBlocker, among others. Pharmacological methods, like EMLA, often demand extended onset times, requiring application up to an hour in advance.^[6]^ This is essential to achieve dermal and epidermal anesthesia, rendering them less suitable for urgent medical cases or in busy pediatric emergency departments. Furthermore, reports suggest that EMLA application may yield cutaneous adverse reactions like erythema, petechiae, or vasoconstriction.^[7]^ Such reactions could exacerbate the difficulty of venipuncture, while the process of removing adhesive patches itself can be distressing. A significant 85.6% of children do not received pain relief interventions during these procedures,^[8]^ highlighting the lack of safe, low-cost, and effective interventions. Safety, especially for children, is of paramount concern. Buzzy is an Food and Drug Administration-approved device shaped like a bee, designed to reduce children’s procedural discomfort through vibration and cooling.^[9]^

While Ballard et al’s meta-analysis^[10]^ spans ages 3 to 18, the analysis did not thoroughly address the experience of needle pain in children under 12, especially those younger than 3. The aim of studies incorporated into their analysis was restricted, with most of the conclusions drawn from individual studies. In recent times, there has been an emergence of numerous randomized controlled trials (RCTs). The present meta-analysis aims to evaluate the safety and efficacy of Buzzy device during needle-related procedures for children under 12 years old. The overarching objective is to provide robust and persuasive evidence in this context.

## 2. Materials and methods

All methods in this study were conducted in accordance with the Preferred Reporting Items for Systematic Reviews and Meta-Analyses guidelines.^[11]^ The pre-registered protocol for this study was published on the Prospero platform (CRD42023447448). Ethical approval was not required due to the study’s nature.

### 2.1. Data source and retrieval

Searches were conducted in the following databases: PubMed, Embase, and Web of Science. The search period extended from the inception of these databases to July 2023. The search strategy utilized a combination of the following keywords: Buzzy, cold, vibration, children. Detailed steps of the literature search are available in the supplementary materials, http://links.lww.com/MD/M143. Besides database searches, the reference lists of all included articles were manually screened to identify any other relevant studies. For specific detailed strategies, please refer to Supplementary Material, http://links.lww.com/MD/M143.

### 2.2. Inclusion criteria

Following the PICOS framework, studies meeting the subsequent criteria were included in this meta-analysis: Participants (P): Children aged 12 years or younger requiring needle-related procedures. Intervention (I): Studies utilizing the Buzzy device as the intervention. Comparison (C): Control groups including no intervention, routine care, or other active control interventions (e.g., distraction cards, VR devices). Outcome assessment (O): Primary outcomes include pain intensity, anxiety intensity, first-attempt success rate, adverse events, and patient/family preferences. Pain and anxiety assessment tools used in the experiments must be validated in the target population. Study Design (S): Only RCTs were considered, irrespective of publication date or status. Systematic reviews, case reports, prospective studies, and original studies without complete text were excluded.

### 2.3. Study selection

The study selection was conducted using EndNote X9.1 software. Two reviewers independently conducted an initial screening of articles based on titles, abstracts, and keywords to identify potentially relevant studies. Articles that met the inclusion criteria underwent a full-text screening to determine final inclusion. In cases of any discrepancies during the aforementioned process, a third reviewer was consulted for resolution.

### 2.4. Data extraction and quality assessment

Two reviewers independently conducted data extraction based on a pre-established data extraction form. The form was initially pilot-tested on three studies and appropriately modified for consistency when extracting information from the remaining studies. Extracted data for each study included research characteristics, sample size, publication year, country, Buzzy usage distance, intervention measures in the control group, and outcomes from all reports. The meta-analysis outcomes comprised pain intensity, anxiety intensity, satisfaction, first puncture attempt rate, and adverse events. Reviewers adhered to the Cochrane Handbook for Systematic Reviews of Interventions, assessing the quality of each study from six aspects: selection bias, performance bias, detection bias, attrition bias, reporting bias, and other biases. Each bias was categorized as high risk, low risk, or unclear risk. The implementation of the blinding technique in non-pharmacological intervention trials posed a challenge. The study categorized “participant and personnel blinding” as high-risk bias for studies using a blank control group and as low risk bias for studies using active control groups Because pain is a subjective experience and self-report is essential. Therefore, self-reported pain and anxiety were assigned an “unclear risk bias.” Discrepancies between the two reviewers were resolved through consensus with a third reviewer.

### 2.5. Statistical analysis

Statistical analysis was conducted using Review Manager 5.4 software and Stata statistical software. Fixed-effects or random-effects models were employed, and 95% confidence intervals (CI) were calculated. For continuous variables, mean differences (MD) or standardized mean differences (SMD) were computed. For binary variables, risk ratios (RR) were calculated. Heterogeneity between studies was assessed using the chi-squared test with a significance level of 0.05. The *I*^2^ statistic was used to quantify heterogeneity. Sensitivity analysis was performed by sequentially excluding individual studies to assess their impact on the overall study. Subgroup meta-analyses were conducted to evaluate levels of heterogeneity. We assessed publication bias through funnel plots and quantitatively examined asymmetry using the Egger’s test.

## 3. Results

### 3.1. Search results

**Figure 1.**
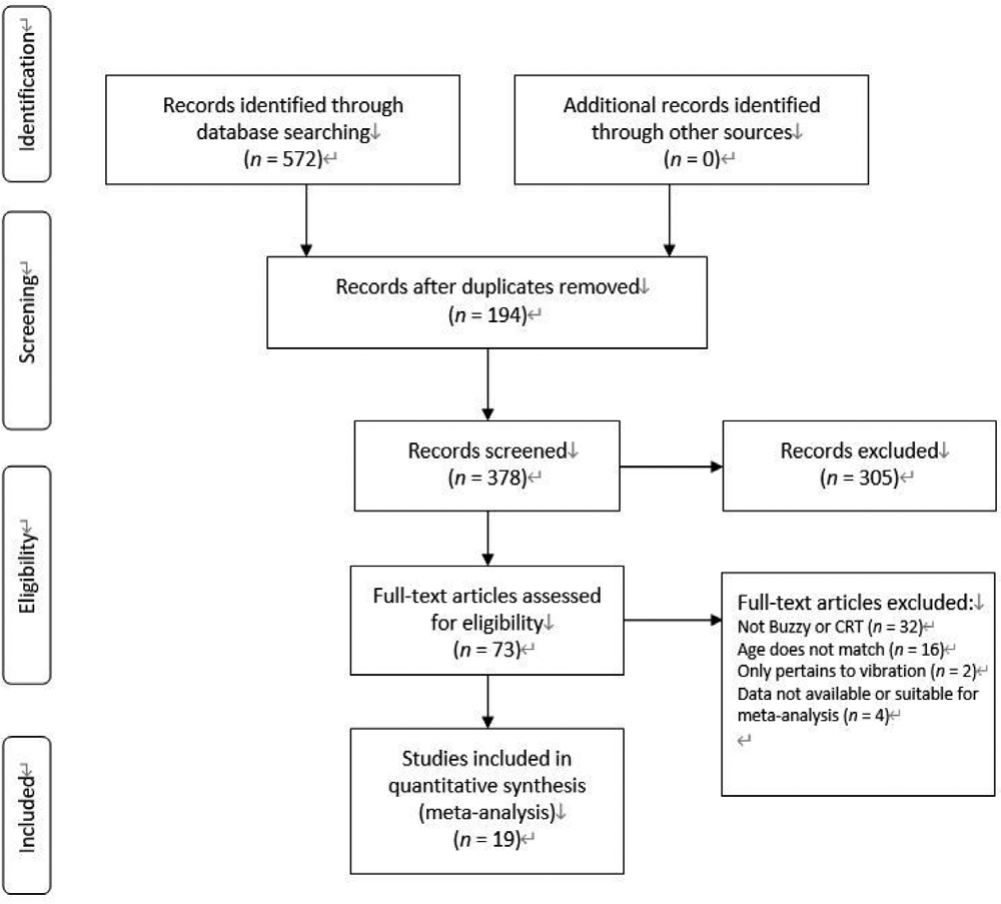
Flow chart of the study selection process.

A total of 572 articles were retrieved from the three databases. After removing 194 duplicates, 378 articles underwent a preliminary screening based on titles and abstracts. There were 73 articles of these underwent full-text examination based on the eligibility criteria. Ultimately, 19 studies involving 2846 participants were deemed eligible for inclusion in this systematic review and meta-analysis. The process of literature inclusion and exclusion is illustrated in Figure 1.

### 3.2. Study characteristics

This review included 19 randomized controlled trials (Table 1), involving a total of 2846 children aged 12 years or younger (Buzzy group n = 1095, Control group n = 1751). The publication years of the included studies ranged from 2012 to 2022. The studies were conducted in several countries, including Turkey,^[12–26]^ Iraq,^[27]^ Lebanon,^[28]^ Saudi Arabia,^[29]^ and France.^[30]^ Of the included studies, 17 studies compared the effects of Buzzy to a no-intervention group,^[12–28]^ 4 studies with ShotBlocker,^[16,19,20,24]^ 3 studies with distraction cards,^[22–24]^ 3 studies involved comparisons with medications: benzocaine gel^[29]^ and EMLA patch,^[30]^ cold spray,^[25]^ 3 studies with VR devices,^[17,23,26]^ 2 studies with bubble-blowing^[18,20]^ and 1 study with whistle blowing.^[27]^ The included studies all focused on needle-related procedures, including venipuncture,^[13,17,18,22,24,27,30]^ vaccination,^[12,15]^ venous catheter insertion,^[14,23,25,26,28]^ insulin injection,^[16]^ intramuscular injection,^[19,20]^ and oral anesthesia.^[21,29]^ In terms of study design, among the 19 randomized studies, four^[12,23,26,29]^ were preregistered RCTs. The remaining 15, though not preregistered, conformed to RCT methodologies, meeting the essential criteria for inclusion in our analysis.

**Table 1:** General description of included studies.

Study	Sample	Study design	Measurement scale	Surgery type	Buzzy distance	Control group	Outcome
Alfatavi Iraq 2022	N = 120 3–6 yr	RCT 3 parallel groups	Wong-Baker Faces Scale Children’s medical fear scale	Venipuncture	5–10 cm	No-treatment Whistling	Pain Anxiety
Siktas Turkey 2022	N = 60 1 yr	RCT 2 parallel groups	FLACC Pain Score	Vaccination	0 cm	No-treatment	Pain
Inal Turkey 2012	N = 12 6–12 yr	RCT 2 parallel groups	Faces Pain Scale-Revised CAPS	Venipuncture	5c m	No-treatment	Pain Anxiety (fear or unease) First-attempt success rate
Canbulat Turkey 2014	N = 176 7–12 yr	RCT 2 parallel groups	Wong-Baker Faces Scale	Venipuncture	5 cm	No-treatment	Pain Anxiety
Canbulat Sabiner Turkey 2014	N = 104 7–12 yr	RCT 2 parallel groups	Wong-Baker Faces Scale Children Fear Scale	Vaccination	5 cm	No-treatment	Pain Anxiety Adverse effects
Moadad Lebanon 2015	N = 48 4–12 yr	RCT 2 parallel groups	Faces Pain Scale-Revised	Venipuncture	5–10 cm	No-treatment	Pain
Sahiner Turkey 2018	N = 60 6–12 yr	RCT 3 parallel groups	Wong-Baker Faces Scale CAPS	Insulin injection	5 cm	No-treatment ShotBlock	Pain
Gerceker Turkey 2018	N = 121 7–12 yr	RCT 3 parallel groups	Wong-Baker Faces Scale	Venipuncture	5 cm	No-treatment VR	Pain
Binay Turkey 2019	N = 96 3–6 yr	RCT 3 parallel groups	Wong-Baker Faces Scale	Venipuncture	5 cm	No-treatment Blowing Soap Bubbles	Pain
Bilgen Turkey 2019	N = 150 7–12 yr	RCT 3 parallel groups	Visual Analog Scale Faces Pain Scale-Revised	Intramuscular injection	3–5 cm	No-treatment ShotBlock	Pain
Yilmaz Turkey 2019	N = 160 5–10 yr	RCT 4 parallel groups	Oucher	Intramuscular injection	3–5 cm	No-treatment ShotBlock Blowing Soap Bubbles	Pain Anxiety
Bilsin Turkey 2019	N = 60 7–12 yr	RCT 2 parallel groups	Wong-Baker Faces Scale	Oral anesthesia	Not mentioned	No-treatment	Pain
Inal Turkey 2017	N = 218 6–12 yr	RCT 4 parallel groups	Wong-Baker Faces Scale	Venipuncture	5–10c m	No-treatment Distracting cards Buzzy + Distracting cards	Pain
AlHareky Saudi Arabia 2021	N = 51 5–12 yr	RCT 2 parallel groups	Visual Analog Scale FLACC Pain Score SEM scale	Oral anesthesia	Not mentioned	20% benzocaine gel	Pain
Bourdier France 2019	N = 607 1.5–6 yr	RCT 2 parallel groups	CHEOPS	Venipuncture	Not mentioned	EMLA	Pain First-attempt success rate Satisfaction
Erdogan Turkey 2021	N = 142 7–12 yr	RCT 4 parallel groups	Visual Analog Scale Wong-Baker Faces Scale Children’s Fear Scale	Venipuncture	3 cm	No-treatment VR Distracting cards	Pain Anxiety
Sivri Turkey 2022	N = 242 9–12 yr	RCT 4 parallel groups	Visual Analog Scale Faces Pain Scale-Revised STAIC	Venipuncture	3–5 cm	No-treatment ShotBlock Distracting cards	Pain Anxiety Satisfaction
Semerci Turkey 2022	N = 161 5–12 yr	RCT 3 parallel groups	Wong-Baker Faces Scale Child Anxiety Statement Scale	Venipuncture	5 cm	No-treatment Cold spray	Pain Anxiety
Yildirim Turkey 2022	N = 150 4–10 yr	RCT 3 parallel groups	Wong-Baker Faces Scale	Venipuncture	5 cm	No-treatment VR	Pain Anxiety First-attempt success rate

Various assessment tools were used to evaluate the level of pain or anxiety (fear, distress, or discomfort) during or after needle-related procedures. These included the Wong-Baker Faces Pain Rating Scale (WB-Faces)^[14,23,25,26,28]^ and the Faces Pain Scale – Revised (FPS-R),^[14,23,25,26,28]^ the FLACC Scale,^[14,23,25,26,28]^ the Oucher Scale,^[14,23,25,26,28]^ and the Children’s Hospital of Eastern Ontario Pain Scale (CHEOPS).^[14,23,25,26,28]^ The WB-Faces, employing a range of facial images from smiling to crying, provides an intuitive visual analog for children to communicate their pain. The FPS-R, an advancement of the original Faces Pain Scale, offers a more refined set of criteria for assessing the nuances of pain intensity. Furthermore, the FLACC Scale, which stands for Face, Legs, Activity, Cry, and Consolability, is utilized for nonverbal patients to assess pain based on observations of behavior. The Oucher Scale combines numerical pain ratings with photographic images for children to indicate their pain levels, catering to a broader age range. The Children’s Hospital of Eastern Ontario Pain Scale (CHEOPS), another behavioral scale, evaluates pain based on both physical and verbal responses to procedural pain. All the adopted measurement scales have undergone validation and are deemed reliable for the target population. When more than one measurement scale or assessor was employed to gauge pain intensity during the procedure, the scale designated as the primary outcome by the study authors was chosen.

### 3.3. Risk of bias

**Figure 2.**
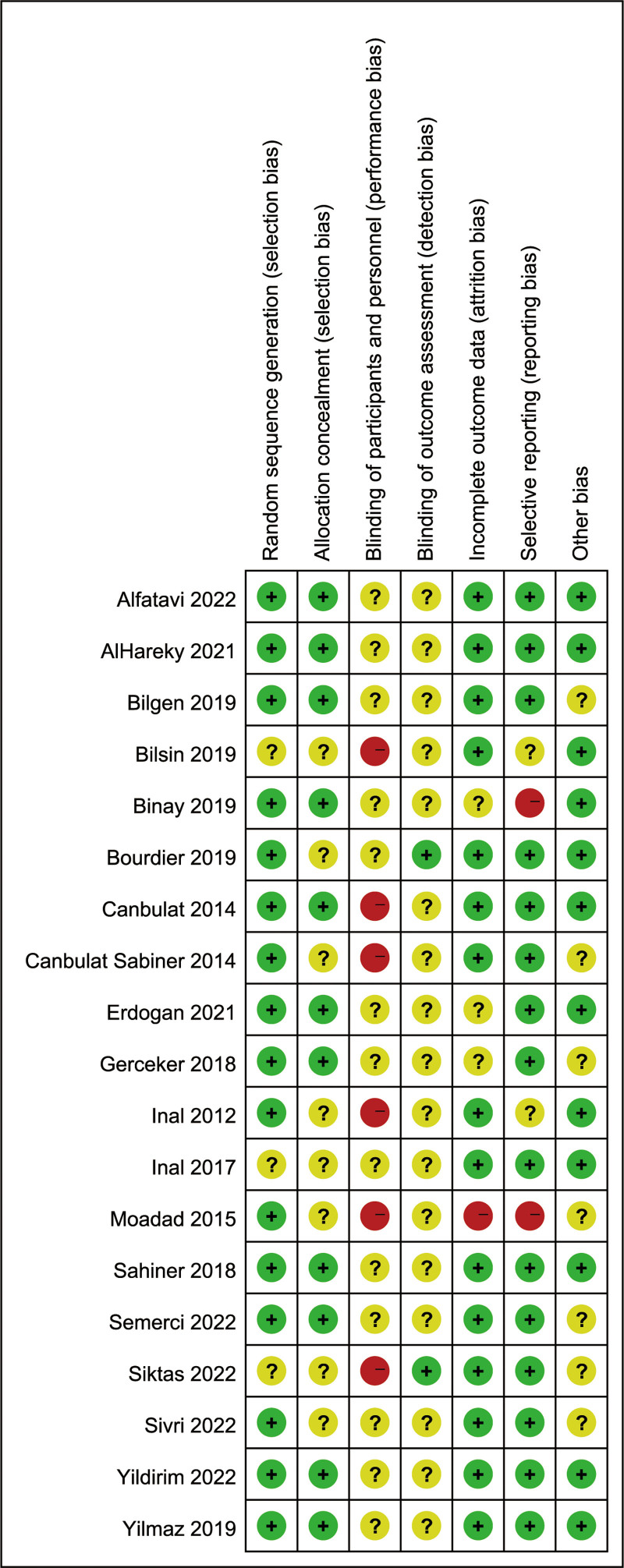
Risk of bias. + = low risk,? = unclear risk, - = high risk.

The results of the risk of bias assessment for all studies are presented in Figure 2. Although all studies claimed to have conducted random allocation, four of them^[12,21,22,24]^ did not explicitly mention the method used for randomization, resulting in an unclear risk of bias. Regarding allocation concealment bias, eight studies did not clearly specify allocation concealment^[12,13,15,22,24,28,30]^ resulting in an unclear risk of bias. In terms of blinding of participants and personnel, six studies^[12–15,21,28]^ only involved a blank control group, resulting in a high risk of bias. The remaining studies included active control groups were assessed as unclear risk of bias. Blinding of outcome assessments is challenging due to the subjective nature of pain and anxiety. Most studies were categorized as unclear risk of bias in this aspect, as self-reported outcomes were the primary basis. Only two studies^[12,30]^ were evaluated as low bias risk due to the employment of observer to assess. One study^[28]^ was evaluated high risk of bias due to incomplete outcome data reporting. Three studies^[17,18,23]^ had varying degrees of participant attrition with insufficient details for proper imputation, resulting in an unclear risk of bias. The remaining studies were categorized as low risk of bias. Two studies^[18,28]^ solely focused on children with successful first-attempt punctures, potentially influencing outcomes and resulting in a high risk of bias. Two studies^[13,21]^ lacked available protocols and were not registered as clinical trials, resulting in an unclear risk of bias. In terms of other biases, seven studies^[12,15,17,24,25]^ were categorized as unclear risk of bias due to reasons such as baseline characteristic imbalances or randomization type.

### 3.4. Meta-analysis

A meta-analysis was conducted for post-puncture pain scores, anxiety scores, first-attempt success rate, adverse events, and patient/family preferences. Studies with a single control group had their results presented descriptively.

### 3.5. Pain intensity

**Figure 3.**
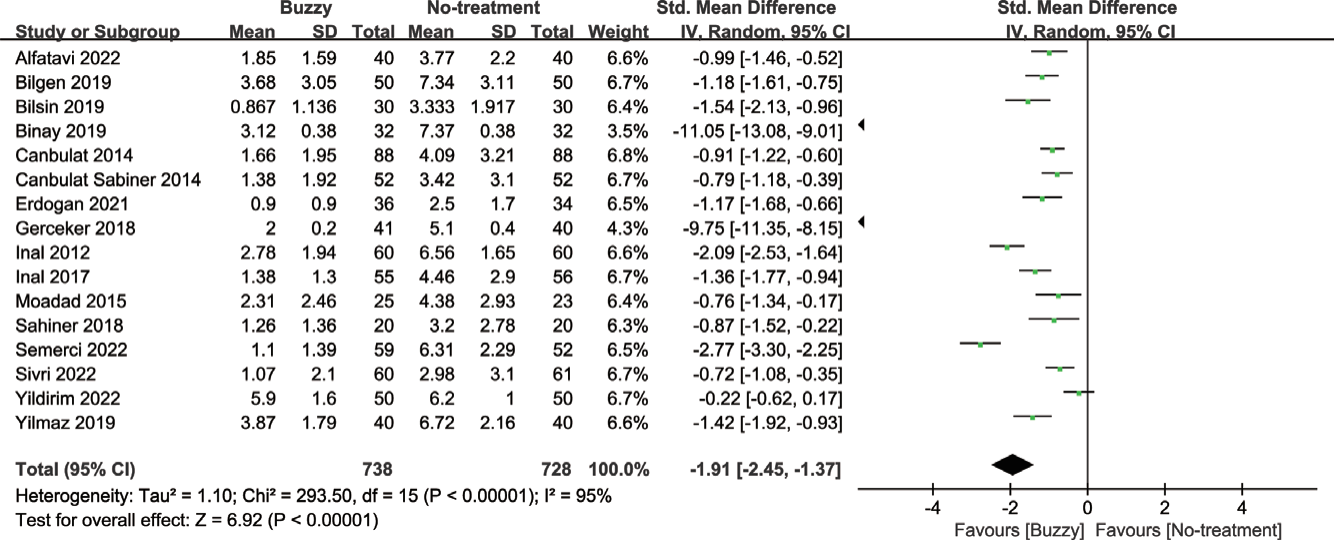
Buzzy vs No-treatment comparator, self-reported pain.

Compared to the no-treatment control group, a total of 16 studies^[13–28]^ analyzed the effects of the Buzzy device on self-reported pain [SMD = −1.91, 95% CI (−2.45 to −1.37), *I*^2^ = 95%, *P* < .00001] the corresponding forest plots can be found in Figure 3. Furthermore, 9 studies^[13,16–18,20,22,23,25,28]^ examined the impact of the Buzzy device on pain reported by parents [SMD = −3.04, 95% CI (−4.09 to −1.99), *I*^2^ = 96%, *P* < .00001, Figure S1, Supplemental Digital Content, http://links.lww.com/MD/M58]. Similarly, 11 studies^[12,13,15–18,20,22,23,25,28]^ analyzed its effects on observer-reported pain [SMD = −2.88, 95% CI (−3.75 to −2.02), *I*^2^ = 96%, *P* < .00001, Figure S2, Supplemental Digital Content, http://links.lww.com/MD/M59]. The results demonstrate a significant effect of the Buzzy device compared to the no-intervention control group for these outcomes.

Compared to the ShotBlock control group, a total of 4 studies^[16,19,20,24]^ analyzed the effects of the Buzzy device on self-reported pain [SMD = −0.29, 95% CI (−0.71 to 0.12), *I*^2^ = 71%, *P* = .17, Figure S3, Supplemental Digital Content, http://links.lww.com/MD/M60]. Additionally, 2 studies^[16,20]^ examined its impact on pain reported by parents [SMD = 0.09, 95% CI (−0.95 to 1.13), *I*^2^ = 86%, *P* = .86, Figure S4, Supplemental Digital Content, http://links.lww.com/MD/M61], and the same studies analyzed its effects on observer-reported pain [SMD = −0.09, 95% CI (−0.66 to 0.48), *I*^2^ = 56%, *P* = .76, Figure S5, Supplemental Digital Content, http://links.lww.com/MD/M62]. The results showed no statistically significant difference between the Buzzy device and the ShotBlock group.

Compared to the Distraction cards control group, a total of 3 studies^[22–24]^ analyzed the effect of the Buzzy device on self-reported pain [SMD = −0.43, 95% CI (−0.90 to 0.05), *I*^2^=76%, *P* = .08, Figure S6, Supplemental Digital Content, http://links.lww.com/MD/M63]. Furthermore,2 studies^[22,23]^ analyzed the effect of the Buzzy device on parent-reported pain [SMD = −0.67, 95% CI (−0.97 to −0.37), *I*^2^=0%, *P* < .0001, Figure S7, Supplemental Digital Content, http://links.lww.com/MD/M64]. Two studies^[22,23]^ analyzed the effect of the Buzzy device on observer-reported pain [SMD = −0.70, 95% CI (−1.00 to −0.40), *I*^2^ = 0%, *P* < .00001, Figure S8, Supplemental Digital Content, http://links.lww.com/MD/M65]. These findings suggest that the Buzzy device significantly reduces parent-reported and observer-reported pain when compared to the Distraction cards control group.

Compared to the VR control group, a total of 3 studies^[17,23,26]^ analyzed the effect of the Buzzy device on self-reported pain [SMD = 0.87, 95% CI (−0.49 to 2.22), *I*^2^ = 96%, *P* = .21, Figure S9, Supplemental Digital Content, http://links.lww.com/MD/M66]. Furthermore 2 studies^[17,23]^ analyzed the effect of the Buzzy device on parent-reported pain [SMD = 1.23, 95% CI (−1.20 to 3.66), *I*^2^ = 98%, *P* = .32, Figure S10, Supplemental Digital Content, http://links.lww.com/MD/M67]. These same studies also analyzed the effect of the Buzzy device on observer-reported pain [SMD = 0.63, 95% CI (−0.61 to 1.86), *I*^2^ = 93%, *P* = .32, Figure S11, Supplemental Digital Content, http://links.lww.com/MD/M68]. The results showed that compared to the VR control group, Buzzy did not have a significant effect on these outcomes.

There were three control interventions related to breathing technique including bubble-blowing^[18,20]^ and whistle-blowing.^[27]^ When comparing Buzzy to the bubble-blowing control group, no significant differences were observed in self-reported [SMD = 1.05, 95% CI (−2.00 to 4.11), *I*^2^ = 98%, *P* = .50, Figure S12, Supplemental Digital Content, http://links.lww.com/MD/M69], parent-reported [SMD = −0.45, 95% CI (−1.15 to 0.25), *I*^2^ = 77%, *P* = .20, Figure S13, Supplemental Digital Content, http://links.lww.com/MD/M70], and observer-reported pain levels[SMD = 0.22, 95% CI (−1.49 to 1.93), *I*^2^ = 96%, *P* = .80, Figure S14, Supplemental Digital Content, http://links.lww.com/MD/M71].However, when comparing Buzzy to the whistle-blowing group, Buzzy significantly reduced self-reported pain [SMD = −0.58, 95% CI (−1.02 to −0.13), *P* = .01, Figure S15, Supplemental Digital Content, http://links.lww.com/MD/M72].

Regarding the pharmaceutical control groups, there were three studies involving interventions with benzocaine gel,^[29]^ EMLA cream,^[30]^ and cooling spray.^[25]^ When comparing Buzzy to the benzocaine gel control group, it significantly reduced self-reported pain levels [SMD = −2.03, 95% CI (−2.72 to −1.35), *P* < .00001, Figure S16, Supplemental Digital Content, http://links.lww.com/MD/M73]. When comparing Buzzy to the EMLA cream control group, it significantly reduced observer-reported pain levels [SMD = −0.52, 95% CI (−0.68 to −0.36), *P* < .00001, Figure S17, Supplemental Digital Content, http://links.lww.com/MD/M74]. Comparing Buzzy to the cooling spray control group, no significant differences were found in self-reported [SMD = −0.30, 95% CI (−0.68 to 0.08), *P* = .13, Figure S18, Supplemental Digital Content, http://links.lww.com/MD/M75], parent-reported [SMD = −0.29, 95% CI (−0.67 to 0.09), *P* = .13, Figure S19, Supplemental Digital Content, http://links.lww.com/MD/M76], and observer-reported pain reduction [SMD = −0.26, 95% CI (−0.64 to 0.12), *P* = .18, Figure S20, Supplemental Digital Content, http://links.lww.com/MD/M77].

### 3.6. Anxiety intensity

**Figure 4.**
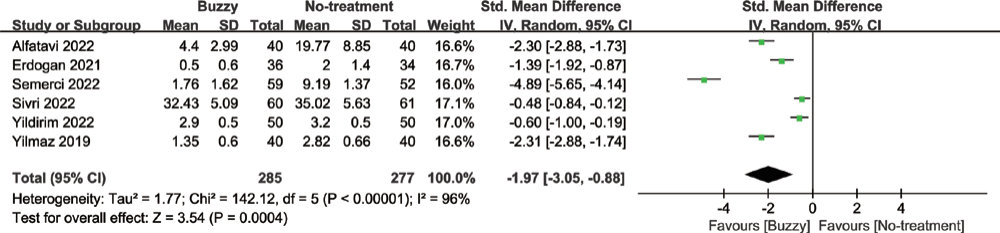
Buzzy vs No-treatment comparator, self-reported anxiety.

In comparison to the no-treatment control group, a total of 6 studies^[20,23–27]^ analyzed the effect of the Buzzy device on self-reported anxiety [SMD = −1.97, 95% CI (−3.05 to −0.88), *I*^2^ = 96%, *P* = .0004], the corresponding forest plots can be found in Figure 4. A total of 5 studies^[13,16,20,23,25]^ analyzed its effect on parent-reported anxiety [SMD = −2.01, 95% CI (−2.93 to −1.08), *I*^2^ = 93%, *P* < .0001, Figure S21, Supplemental Digital Content, http://links.lww.com/MD/M78], and seven studies^[13–16,20,23,25]^ considered its impact on observer-reported anxiety [SMD = −1.92, 95% CI (−2.64 to −1.19), *I*^2^ = 94%, *P* < .00001, Figure S22, Supplemental Digital Content, http://links.lww.com/MD/M79]. The results demonstrate a significant effect of the Buzzy device compared to the no-intervention control group for these outcomes.

In comparison to the Shotblock control group, a total of 2 studies^[20,24]^ evaluated the effect of the Buzzy device on self-reported anxiety [SMD = −0.27, 95% CI (−0.77 to 0.24), *I*^2^=68%, *P* = .30, Figure S23, Supplemental Digital Content, http://links.lww.com/MD/M80]. Two studies^[16,20]^ analyzed the effect of the Buzzy device on parent-reported anxiety [SMD = 0.09, 95% CI (−1.17 to 1.34), *I*^2^=90%, *P* = .89, Figure S24, Supplemental Digital Content, http://links.lww.com/MD/M113]. Two studies^[16,20]^ analyzed the effect of the Buzzy device on observer-reported anxiety [SMD = 0.05, 95% CI (−0.79 to 0.88), *I*^2^ = 79%, *P* = .91, Figure S25, Supplemental Digital Content, http://links.lww.com/MD/M114]. The results suggest that Buzzy did not exhibit a significant effect on these outcomes compared to the Shotblock control group.

In comparison to the Distraction cards control group, a total of 2 studies^[23,24]^ analyzed the effect of the Buzzy device on self-reported anxiety [SMD = −0.38, 95% CI (−1.39 to 0.63), *I*^2^ = 91%, *P* = .46, Figure S26, Supplemental Digital Content, http://links.lww.com/MD/M115]. Additionally, one study^[23]^ explored the effect of the Buzzy device on parent-reported [SMD = −0.96, 95% CI (−1.46 to −0.47), *P* = .0001, Figure S27, Supplemental Digital Content, http://links.lww.com/MD/M116] and observer-reported anxiety [SMD = −0.91, 95% CI (−1.40 to −0.42), *P* = .0003, Figure S28, Supplemental Digital Content, http://links.lww.com/MD/M117]. The results indicate that the Buzzy device significantly reduced anxiety levels reported by parents and observers, compared to the Distraction cards control group.

In comparison to the VR control group, a total of 2 studies^[23,26]^ analyzed the effect of the Buzzy device on self-reported anxiety [SMD = −0.47, 95% CI (−0.77 to −0.17), *I*^2^ = 0%, *P* = .002, Figure S29, Supplemental Digital Content, http://links.lww.com/MD/M118]. Additionally, one study^[23]^ assessed the effect of the Buzzy device on parent-reported [SMD = −0.42, 95% CI (−0.88 to 0.05), *P* = .08, Figure S30, Supplemental Digital Content, http://links.lww.com/MD/M119] and observer-reported anxiety [SMD = −0.28, 95% CI (−0.74 to 0.18), *P* = .24, Figure S31, Supplemental Digital Content, http://links.lww.com/MD/M120]. The results show that the Buzzy device significantly reduced anxiety levels reported by self-reported, compared to the VR control group.

When compared to the bubble-blowing,^[20]^ the Buzzy device significantly reduced self-reported anxiety [SMD = −0.87, 95% CI (−1.33 to −0.41), *P* = .0002, Figure S32, Supplemental Digital Content, http://links.lww.com/MD/M121], parent-reported anxiety [MD = −1.00, 95% CI (−1.47 to −0.53), *P* < .0001, Figure S33, Supplemental Digital Content, http://links.lww.com/MD/M124], and observer-reported anxiety [SMD = −0.65, 95% CI (−1.10 to −0.20), *P* = .005, Figure S34, Supplemental Digital Content, http://links.lww.com/MD/M126]. In contrast to the whistle control group,^[27]^ Buzzy significantly reduced self-reported anxiety [SMD = −2.30, 95% CI (−2.88 to −1.73), *P* < .0001, Figure S35, Supplemental Digital Content, http://links.lww.com/MD/M128].

Compared to the cold spray^[25]^ control group, there were no significant differences in reducing self-reported anxiety [SMD = −0.12, 95% CI (−0.49 to 0.26), *P* = .54, Figure S36, Supplemental Digital Content, http://links.lww.com/MD/M129], parent-reported anxiety [SMD = −0.03, 95% CI (−0.41 to 0.35), *P* = .88, Figure S37, Supplemental Digital Content, http://links.lww.com/MD/M132], and observer-reported anxiety [SMD = -0.12, 95% CI (-0.49 to 0.26), *P* = .54, Figure S38, Supplemental Digital Content, http://links.lww.com/MD/M133] between the Buzzy device and the cold spray.

### 3.7. Success rate of first puncture attempt

**Figure 5.**
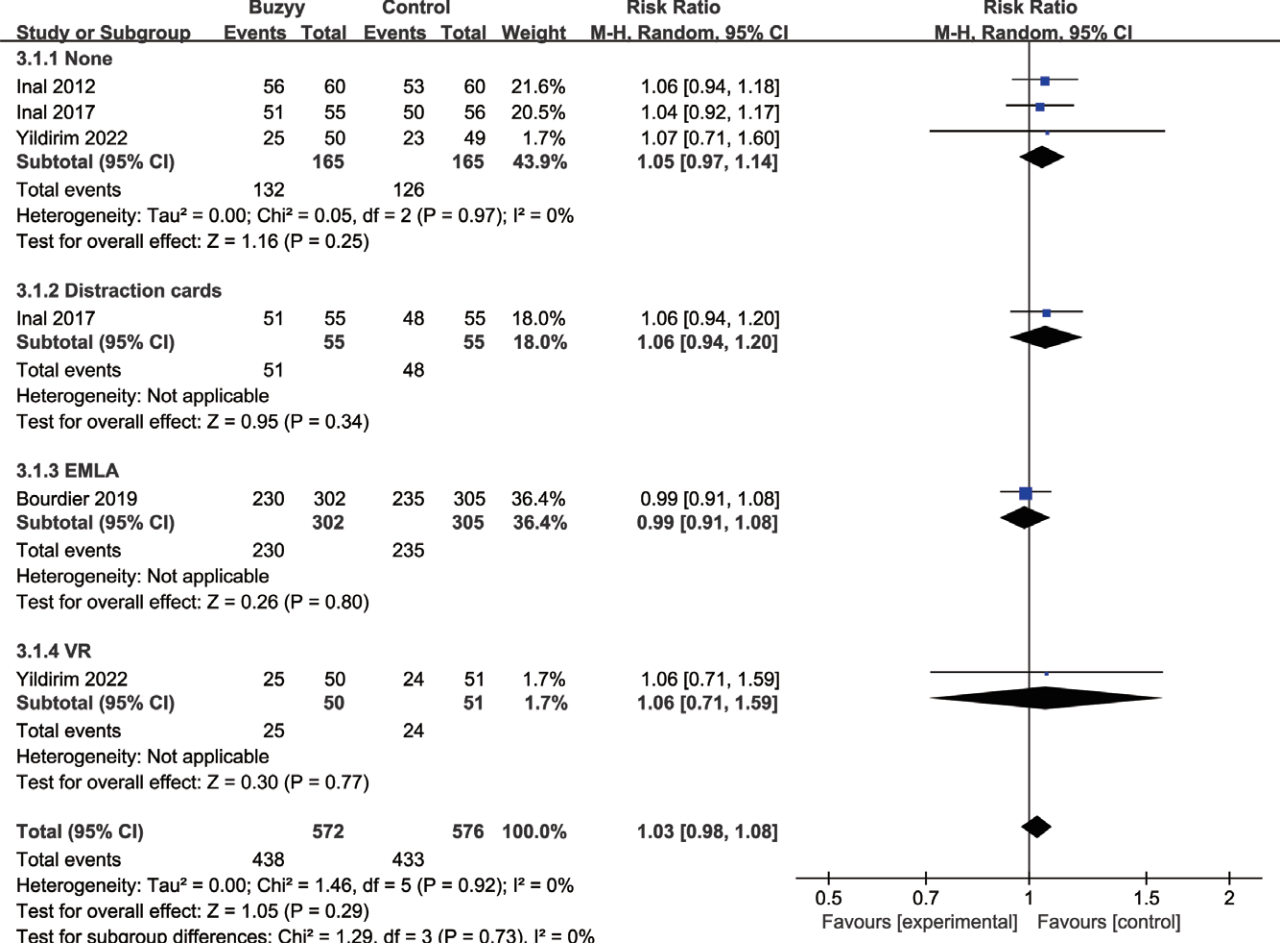
Success of the procedure at first attempt.

The success rate of the first puncture attempt was analyzed across 4 studies^[13,22,26,30]^ involving control groups with no-treatment control group, distraction card group, EMLA group, and VR group. Compared to these intervention measures, the Buzzy device did not show a significant impact on the success rate of the first puncture attempt, with an overall effect of [RR = 1.03, 95% CI (0.98 to 1.08), *I*^2^ = 0%, *P* = .29] (Fig. 5).

### 3.8. Satisfaction/preference

In one study,^[24]^ children’s preferences for different intervention measures were discussed. The study’s conclusion revealed that a higher proportion of children felt “very good” in the Buzzy and Distraction groups than in the ShotBlock and control groups. Moreover, a greater proportion of children in the control group felt either “moderately” or “poorly” compared to the other groups. However, another study^[30]^ showed that an investigation showed that family members were more willing to use the EMLA method compared to Buzzy.

### 3.9. Adverse events

One study^[15]^ that involved 104 children (Buzzy = 52, None = 52) reported adverse events as an outcome measure. However, no adverse events were observed in either the Buzzy group or the no intervention group.

### 3.10. Publication bias

The Egger’s test was conducted to assess publication bias for datasets incorporating 10 or more studies. The outcomes showed that compared to the control group with no treatment, self-reported pain scores exhibited publication bias (Egger’s test *P* = .013). In contrast, observer-reported scores showed no evident publication bias (Egger’s test *P* = .119).

### 3.11. Subgroup analysis

**Figure 6.**
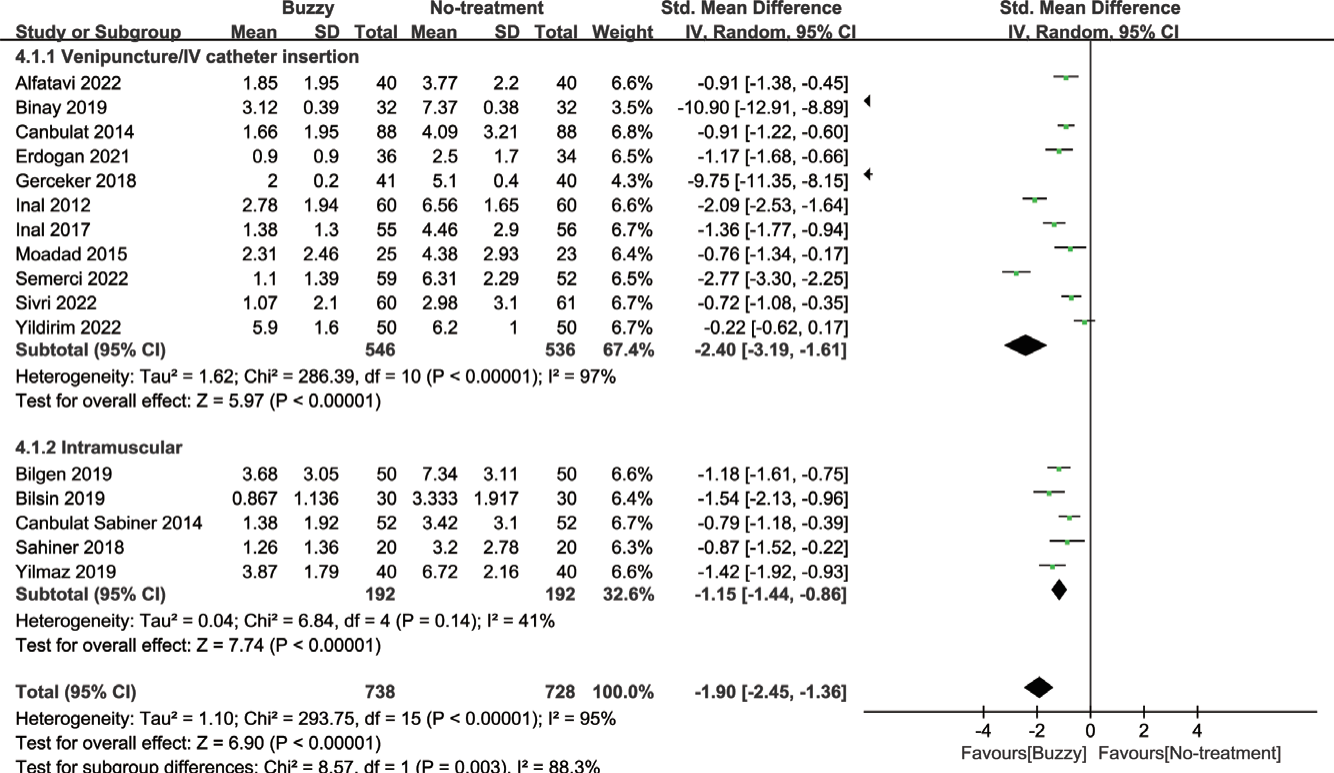
Subgroup analyses of buzzy device efficacy in pain relief.

Subgroup analyses on procedure type indicated that Buzzy exerted better pain relief than no intervention during both venipuncture and intramuscular injection (Fig. 6). Subgroup analyses based on age showed that Buzzy demonstrated favorable analgesic effects in both children over and under 6 years of age (Figures S39 and S40, Supplemental Digital Content, http://links.lww.com/MD/M135, http://links.lww.com/MD/M136). Based on the distance between the Buzzy device and the site of puncture, participants were divided into three groups. The results showed that all three groups demonstrated a significant reduction in self-reported pain (Figure S41, Supplemental Digital Content, http://links.lww.com/MD/M138). However, the group with the recommended 5cm distance [SMD = -3.11, 95% CI (-4.26 to -1.96), *I*^2^ = 97%, *P* < .00001, Figure S41, Supplemental Digital Content, http://links.lww.com/MD/M138] exhibited better outcomes compared to the other two groups. Subgroup analyses based on pain assessment scales showed that Buzzy was effective in improving pain responses regardless of the scale used (Figure S42, Supplemental Digital Content, http://links.lww.com/MD/M140).

## 4. Discussion

The objective of this meta-analysis was to evaluate the effectiveness and safety of the Buzzy device in pediatric needle procedures. Through a comprehensive review and analysis of existing research, a total of 19 randomized controlled trials were included, assessing the impact of the Buzzy device on pain intensity, anxiety intensity, first success rate of puncture attempt, patient attitudes, and adverse events. The results indicate that compared to the no intervention group, the Buzzy device significantly diminishes pain and anxiety levels associated with needle procedures reported by children under the age of twelve, as reported by the children themselves, parents, and observers. Buzzy’s pain-alleviating effect surpasses whistle-blowing, benzocaine gel, and EMLA when self-reported. When reported by parents and observers, it is more effective than the Distraction Cards group. Its anxiety-alleviating prowess, as self-reported, outdoes the VR, bubble-blowing, and whistle-blowing groups. According to parents and observers, Buzzy’s efficacy exceeds the Distraction Cards and bubble-blowing groups. The summarized first puncture attempt success rate did not show any significant difference compared to other intervention measures, and none of the included studies reported adverse reactions.

Although the Buzzy device demonstrated significant effects in reducing pain and anxiety across the included studies, we observed notable heterogeneity in the results, prompting an investigation into its potential sources. Considering that 89.5% of the studies involved no-intervention control groups, we explored the impact of various factors in this specific subgroup, including age, type of needle procedure, and distance from the puncture site. These analyses revealed that the Buzzy device consistently showed effectiveness across different age groups and procedural contexts. However, the persistence of high heterogeneity suggests the presence of other influential variables not fully captured in this review. The sources of this heterogeneity could stem from the diversity in clinical settings, variations in study designs, and the subjective nature of pain assessment tools. Notably, the subgroup analysis based on the distance of operation show that the recommended 5cm operating distance of the Buzzy device outperformed both shorter and longer distances, underscoring the importance of adhering to standardized protocols. Variations in the types of medication used in procedures, such as those in intramuscular injections, might also contribute to the observed differences in pain outcomes. This discrepancy may arise from medications used in intramuscular injections, like penicillin, potentially causing greater tissue pain at the injection site.^[31]^

To address these disparities, future research should include stricter study designs, detailed protocol standardization, uniform pain assessment tools, and consideration of other potential confounding factors, such as the psychological state of children, procedural anxiety, and cultural perceptions of pain. Methodological improvements of this nature could elucidate the fundamental causes of heterogeneity, enhance the reliability and universality of the findings, and ultimately aid in developing personalized pain management strategies for pediatric patients undergoing needle procedures.

It is noteworthy that there is currently no research analyzing whether the freezing function of the Buzzy device might impact the results of blood collection. Although the

purpose of this study was to investigate effective interventions to alleviate needle pain and anxiety in children, ensuring the accuracy of medical procedures is also equally crucial. The accuracy of results was more meaningful than the search for effective interventions. Furthermore, the bee-shaped design of the Buzzy device might induce subjective discomfort in patients. Altering the appearance to appeal to the preferences of young children could potentially mitigate the sensation of pain.^[32]^ A study^[22]^ showed that the combined use of the Buzzy device and distraction cards might surpass the effectiveness of the sole use of the Buzzy device. However, there is a limited number of studies exploring this intervention, and a meta-analysis was not conducted. Future research could focus on investigating the effects of noninvasive, non-pharmacological interventions when used in combination. This would be beneficial in identifying more effective methods to reduce pain and anxiety associated with needling in children. Patient satisfaction is noteworthy, with many expressing a willingness to utilize the Buzzy device again for pain relief. Nevertheless, the limited number of studies on adverse events and satisfaction outcomes, and the concentration of trial locations in a single region, indicate potential constraints. Therefore, future research efforts should focus on more rigorous and extensive studies to ascertain the efficacy of the Buzzy device in needle procedures.

## 5. Limitations

There are three potential limitations in this systematic review. First, a limited number of studies included adverse events and satisfaction as outcome measures, which resulted in a small pool of studies for meta-analysis. Second, most trials in our review were conducted in Turkey, possibly limiting the wider applicability of our results. Third, due to the nature of physical intervention trials, blinding was not feasible in all instances.. Although we have included all relevant studies, future research should aim to provide more high-quality randomized controlled trials to further support our findings.

## 6. Conclusion

In conclusion, Buzzy device demonstrates promising effects in reducing pain and anxiety during pediatric needle procedures and will not impair the puncture procedure. Moreover, the method has the advantages of safety, high patient satisfaction, and the absence of significant adverse events. Nevertheless, further rigorous and large-scale research is required to establish the efficacy of the Buzzy device in needle procedures.

## Author contributions

**Conceptualization:** Faguang Jin.

**Data curation:** Faguang Jin.

**Investigation:** Maomao Qi.

**Methodology:** Faguang Jin, Xiaofang Wang.

**Software:** Faguang Jin, Wenhua Zhang.

**Writing – original draft:** Faguang Jin, Xiaofang Wang, Yongfeng Zhang.

**Writing – review & editing:** Faguang Jin, Yongfeng Zhang.

## Supplementary Material

**Figure:**
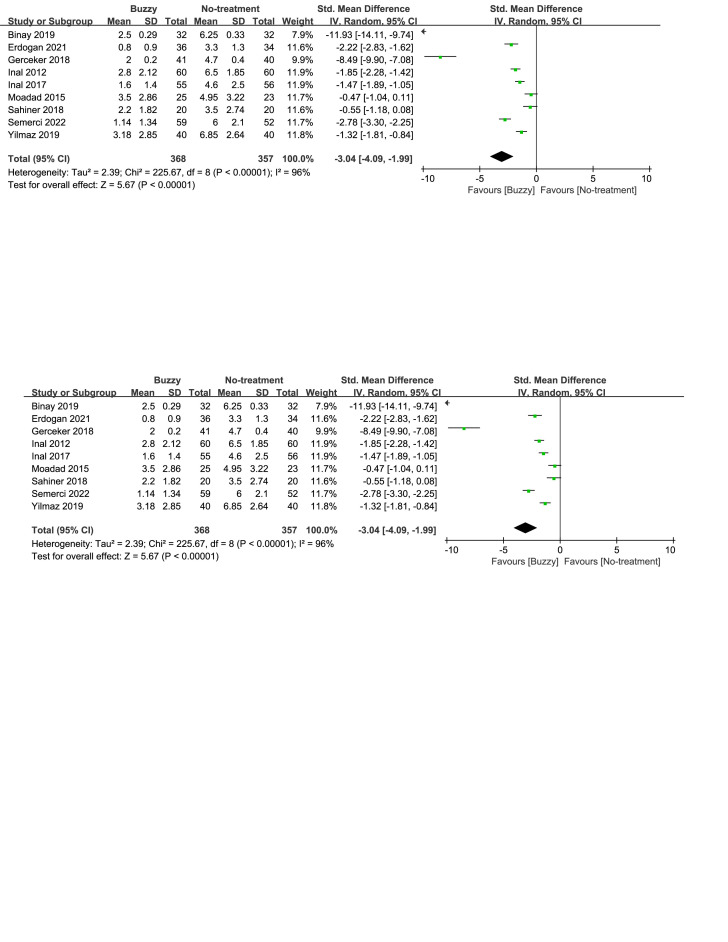


**Figure:**
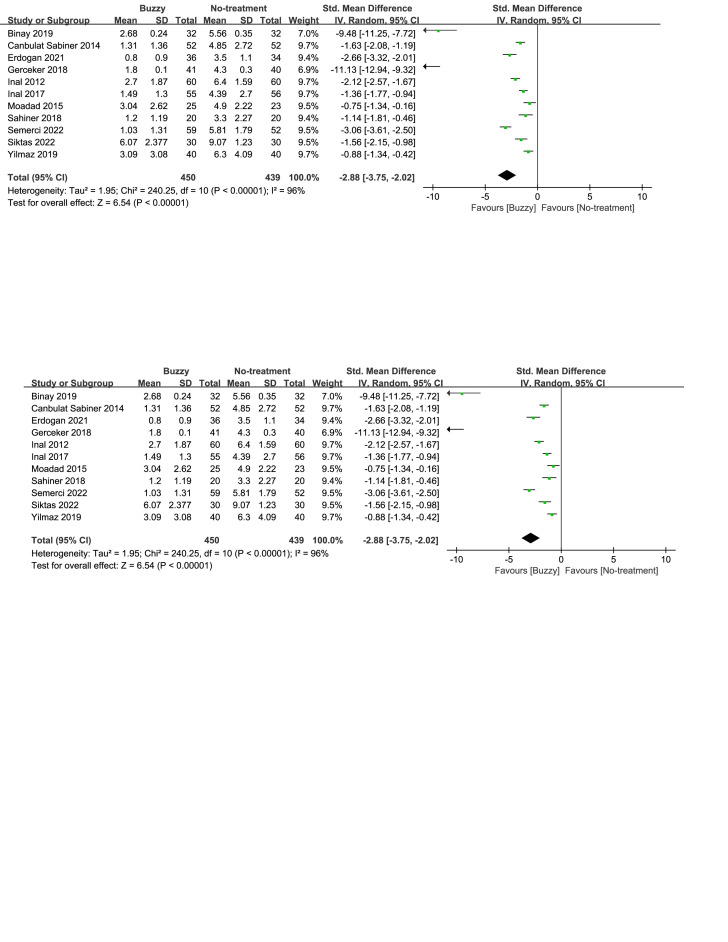


**Figure:**
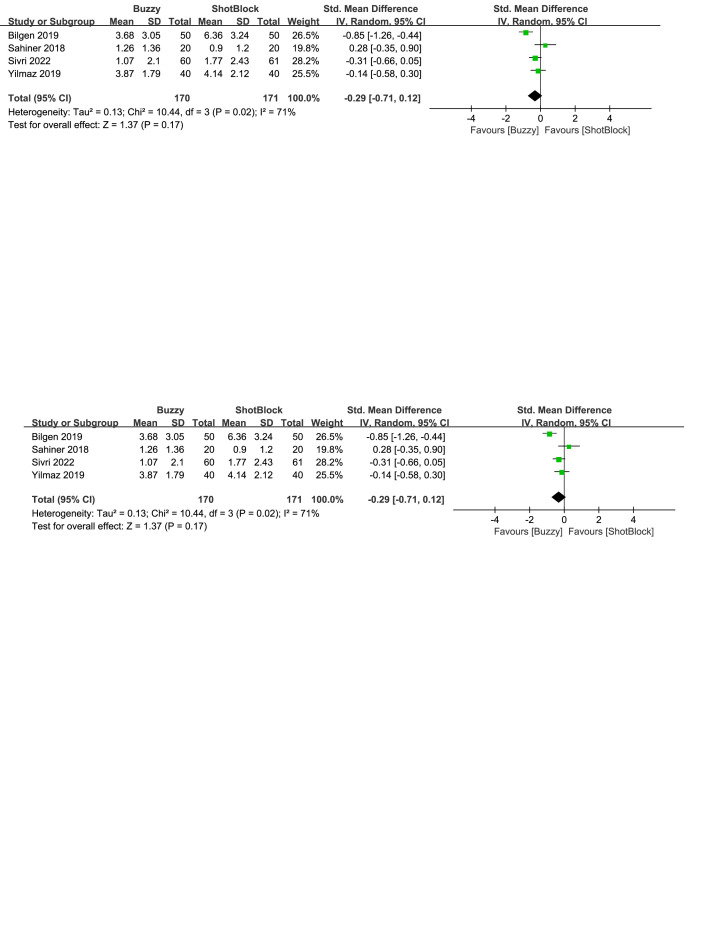


**Figure:**
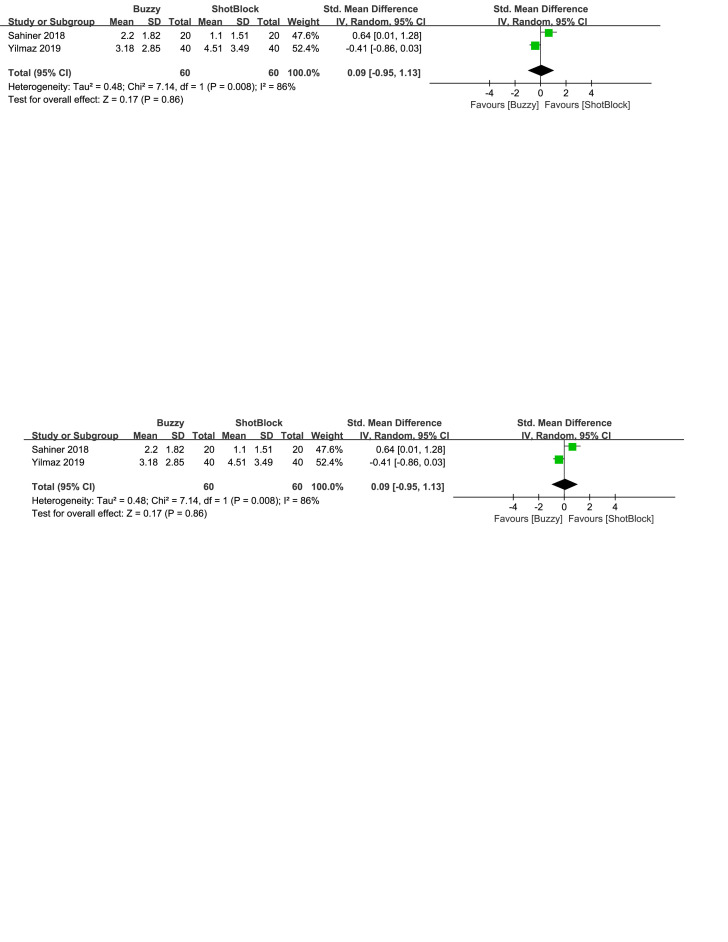


**Figure:**
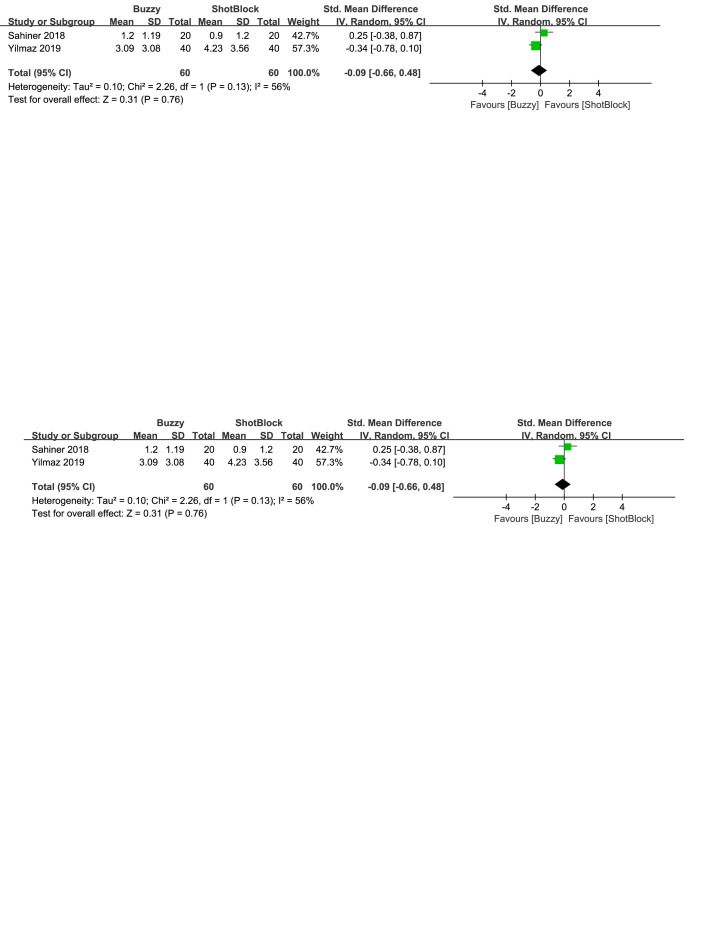


**Figure:**
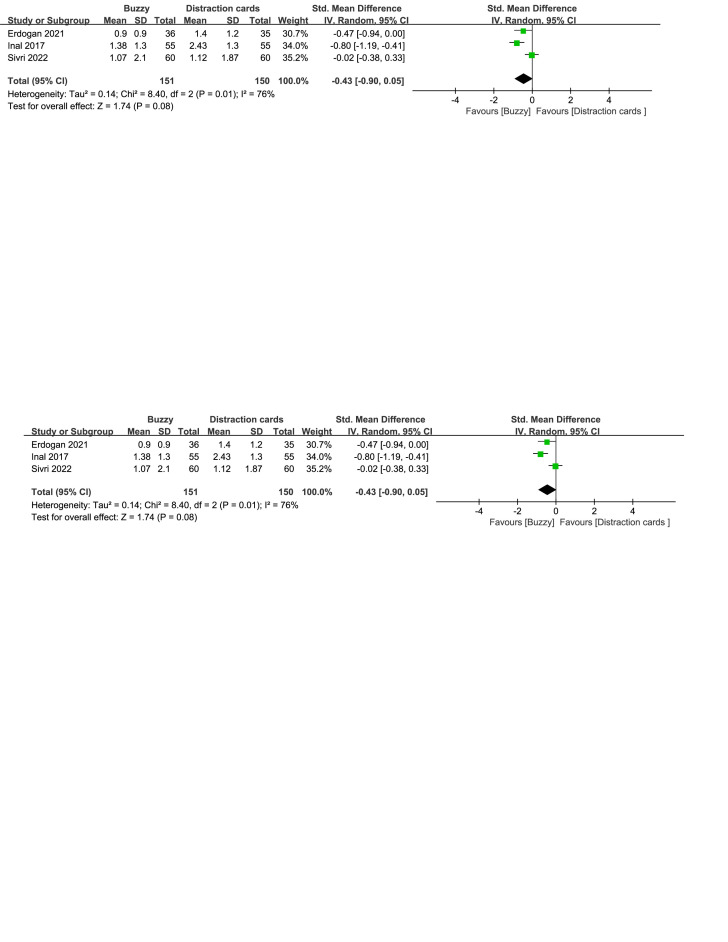


**Figure:**
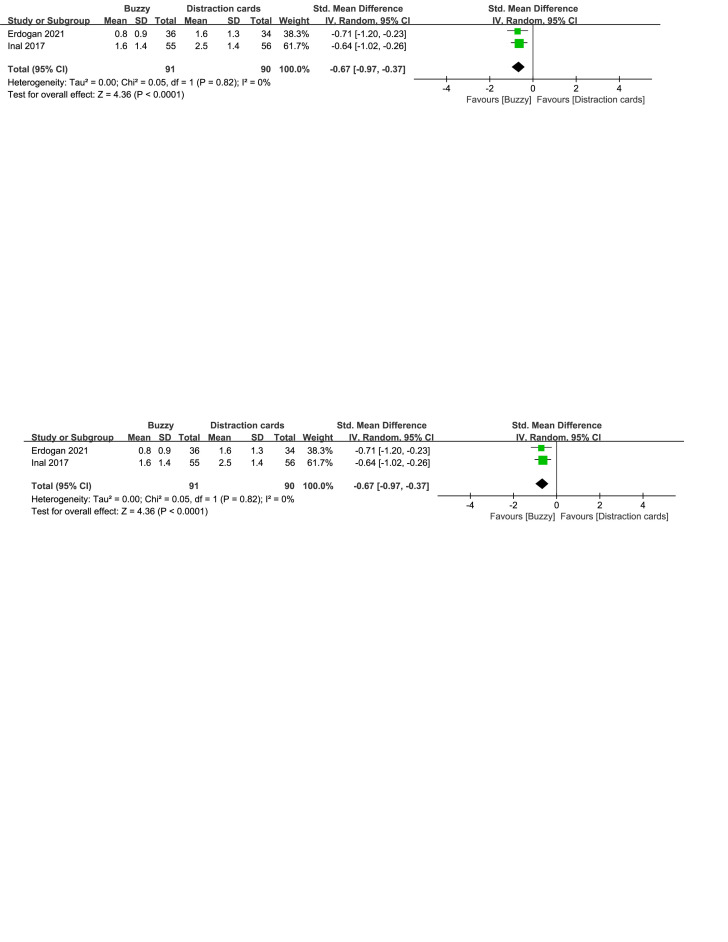


**Figure:**
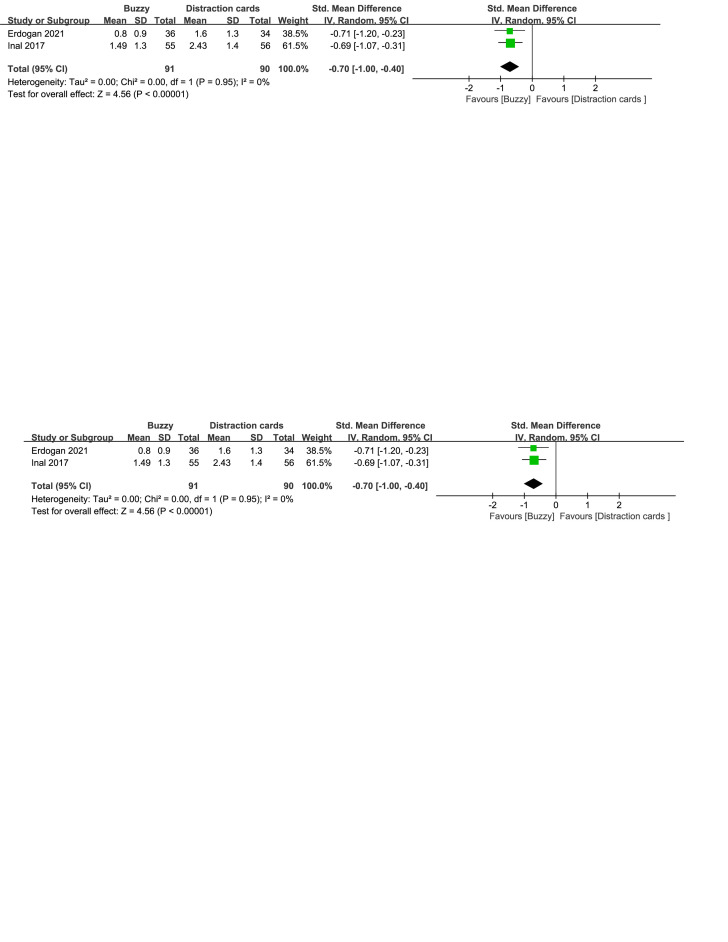


**Figure:**
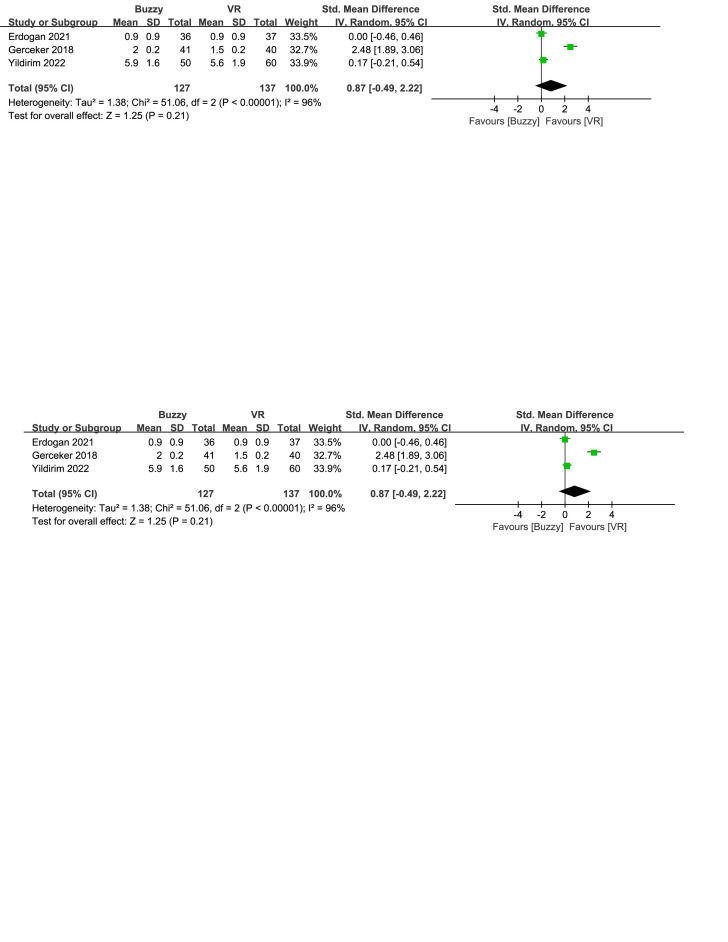


**Figure:**
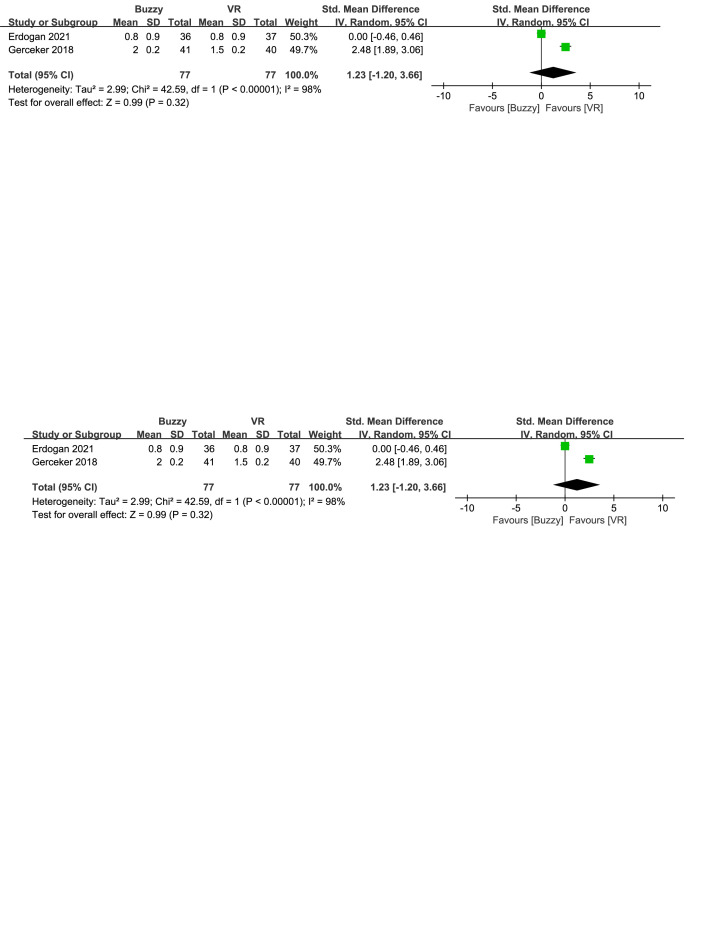


**Figure:**
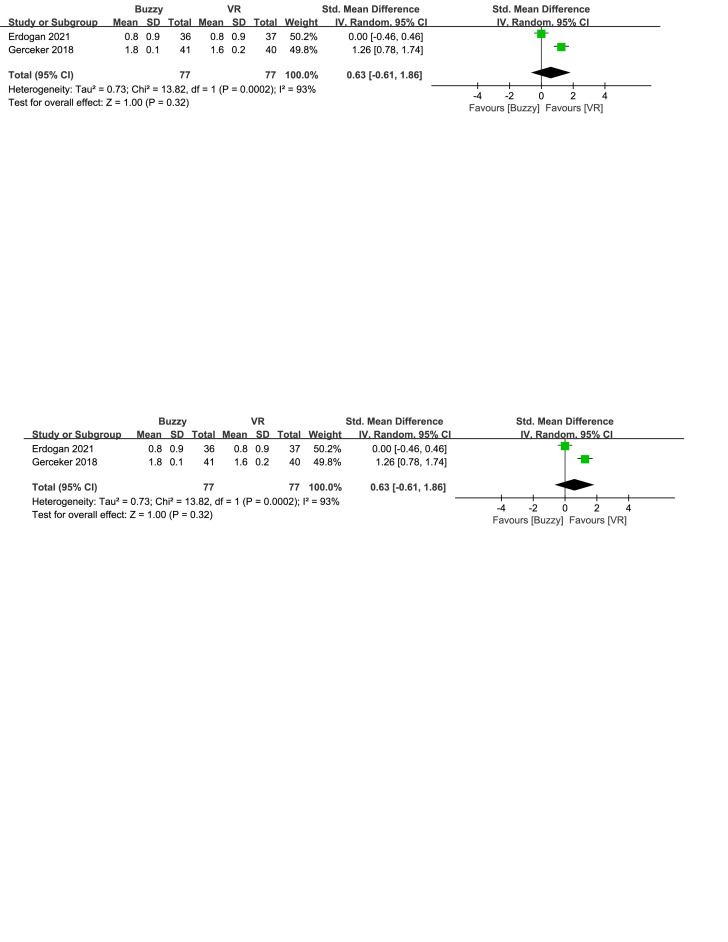


**Figure:**
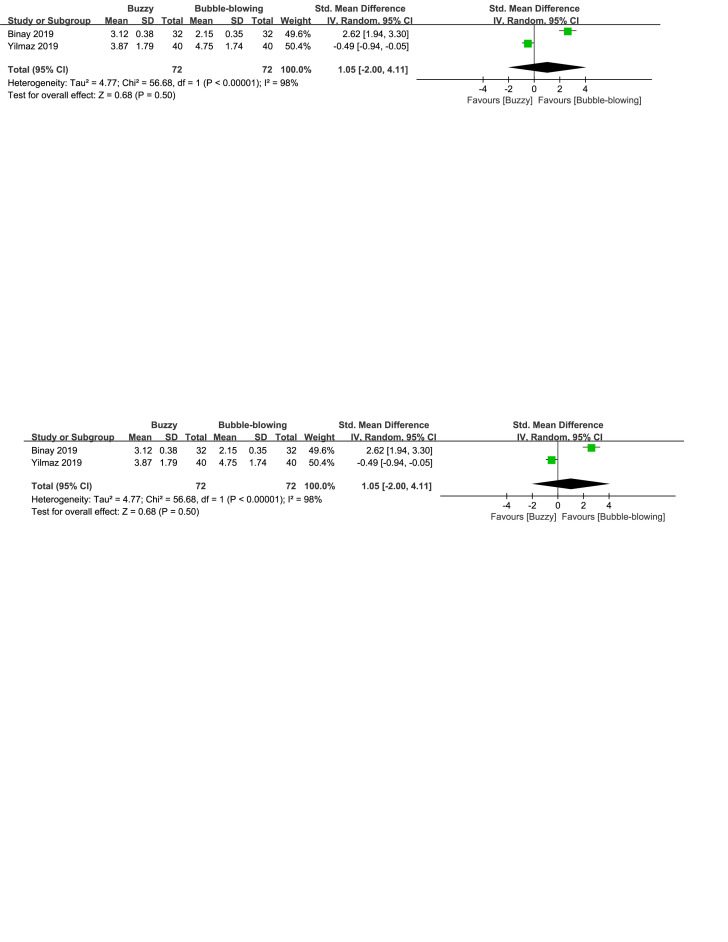


**Figure:**
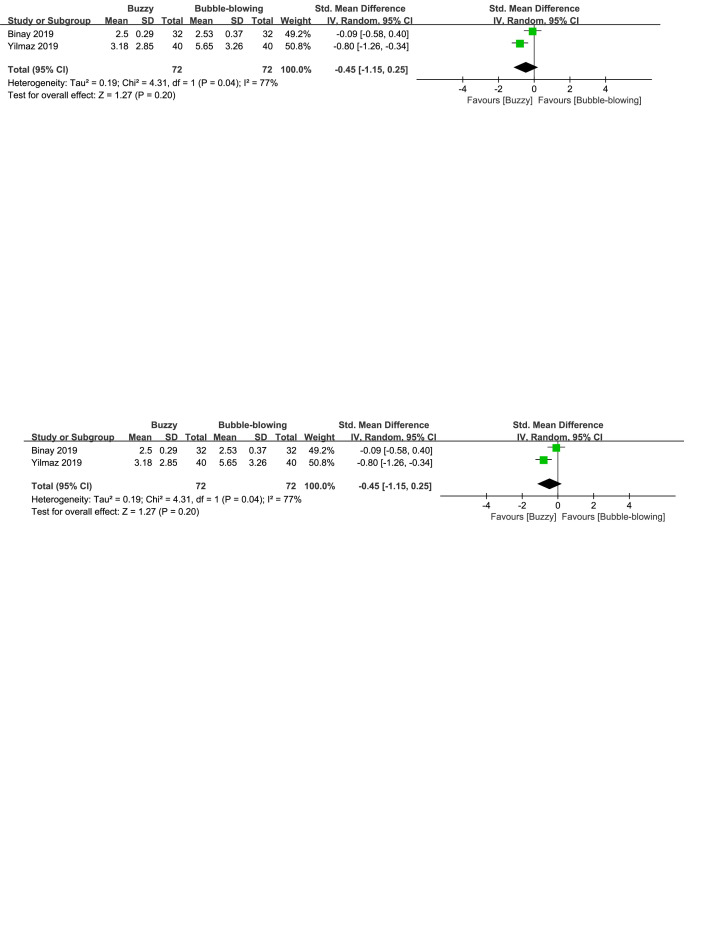


**Figure:**
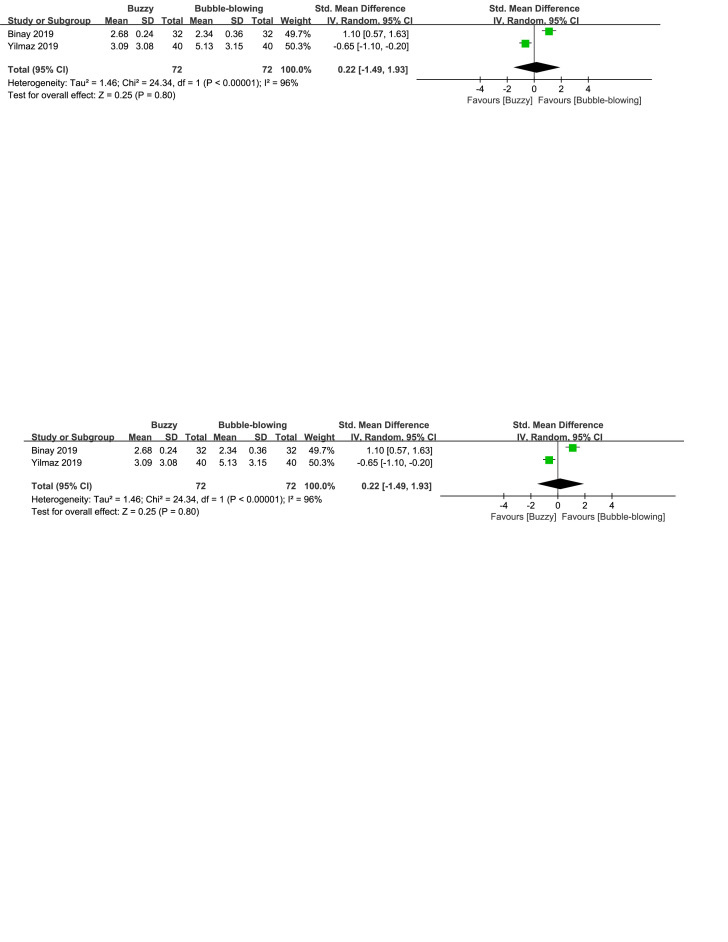


**Figure:**
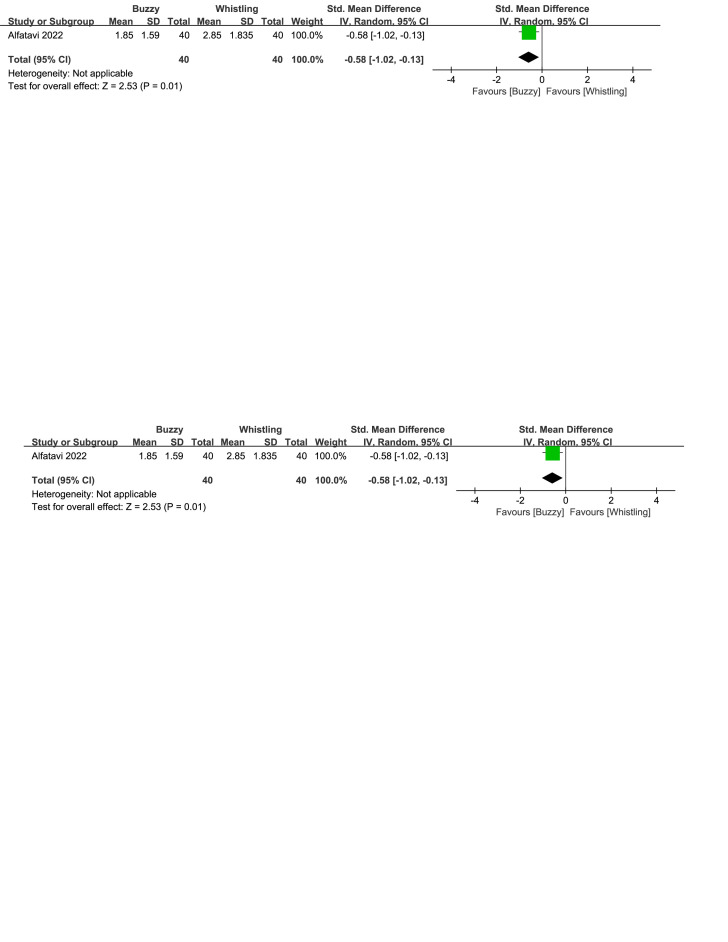


**Figure:**
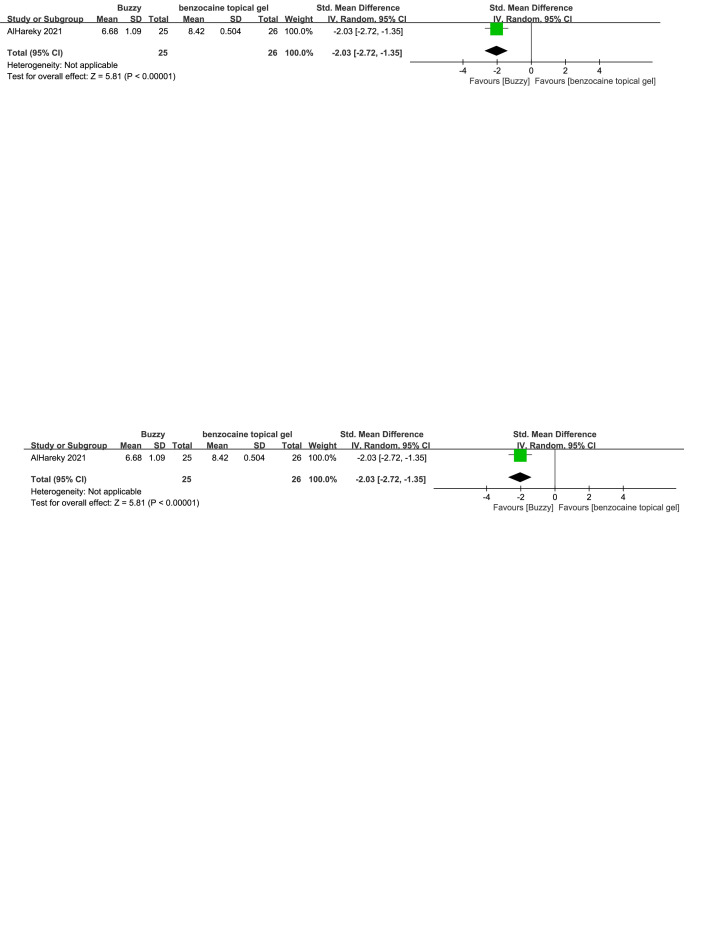


**Figure:**
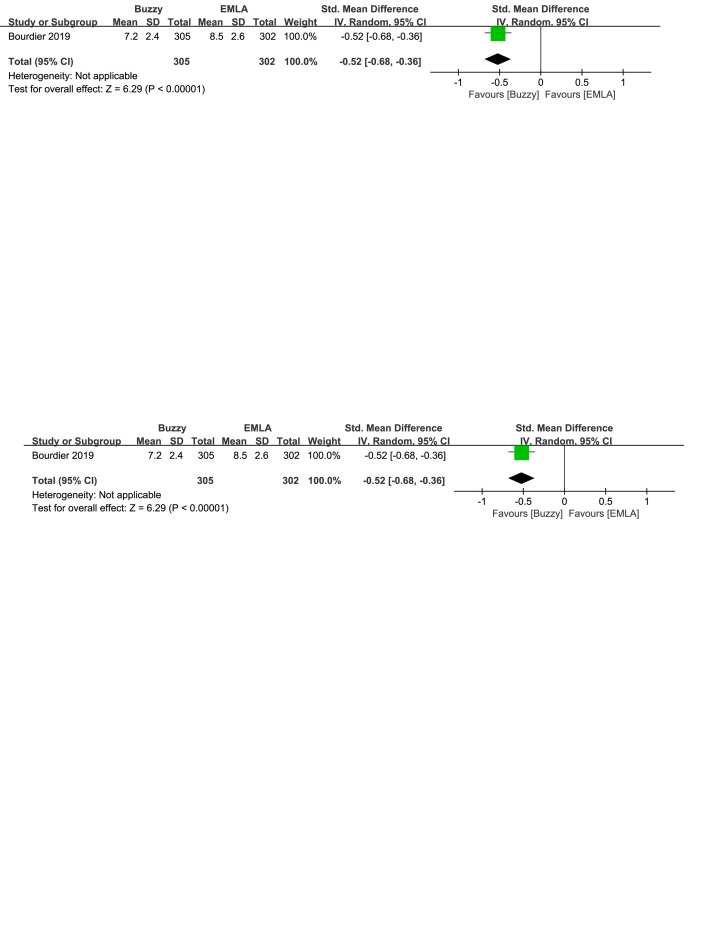


**Figure:**
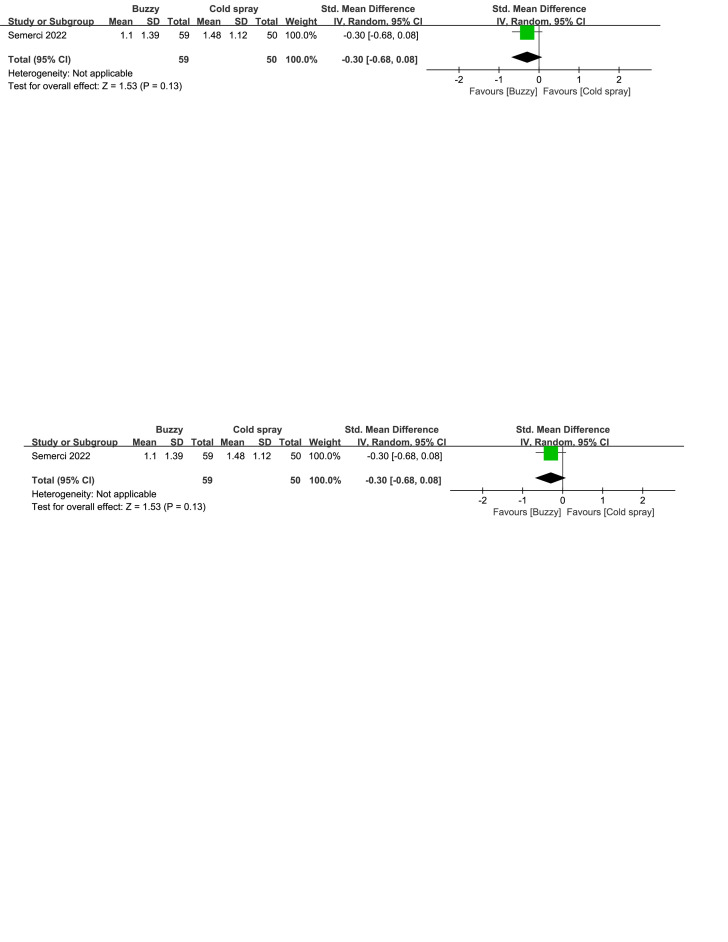


**Figure:**
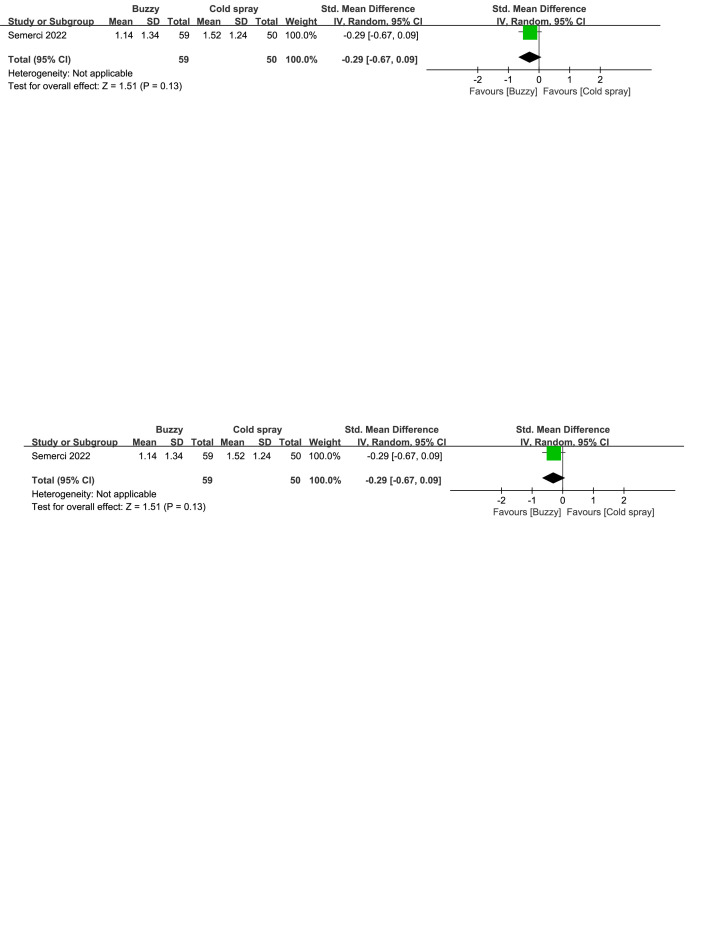


**Figure:**
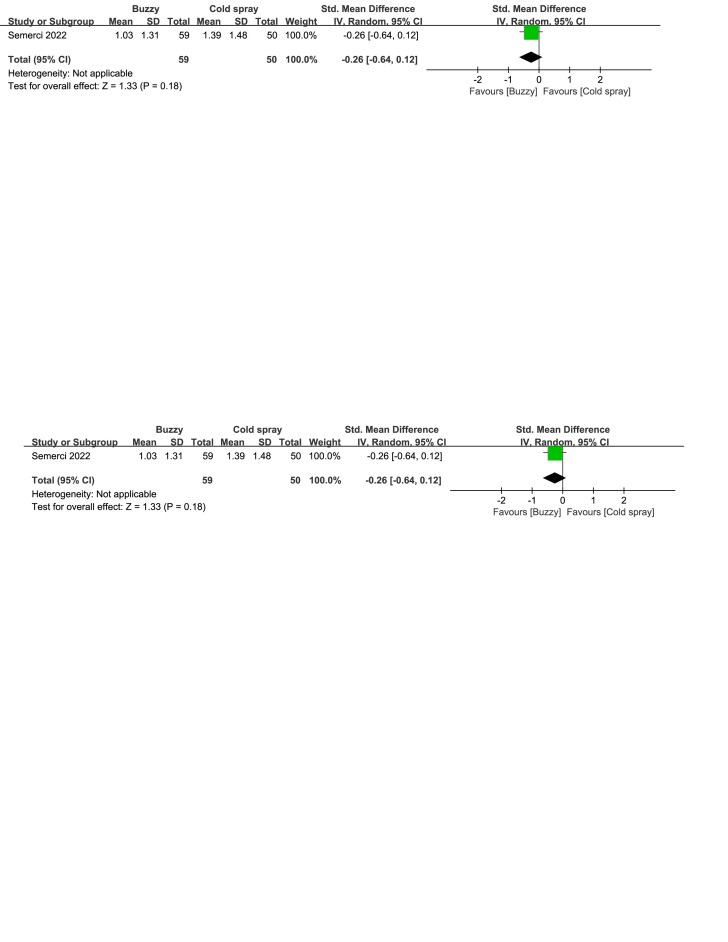


**Figure:**
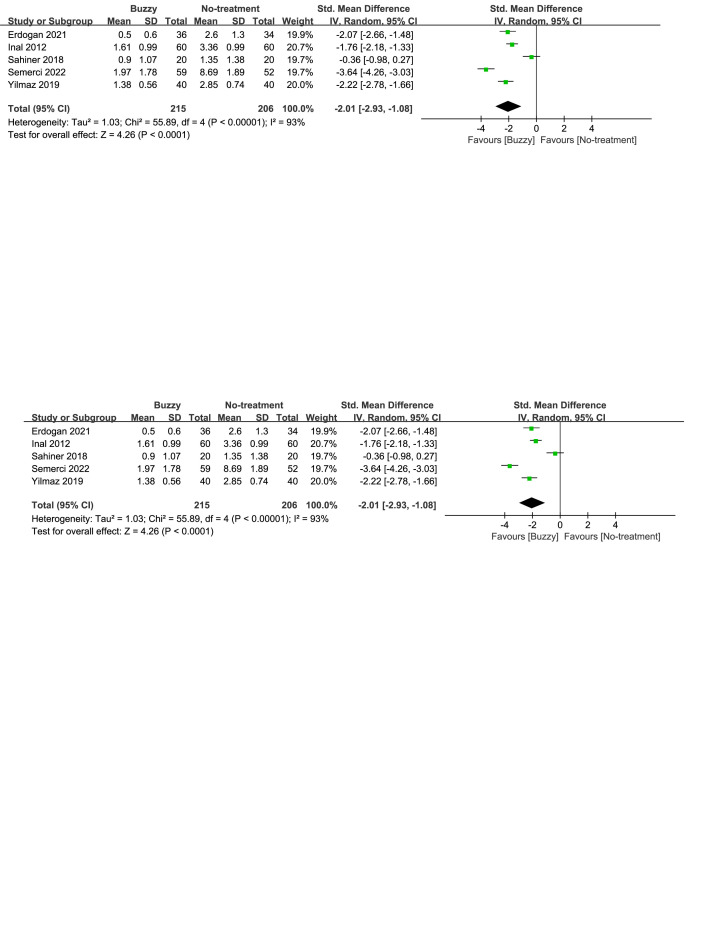


**Figure:**
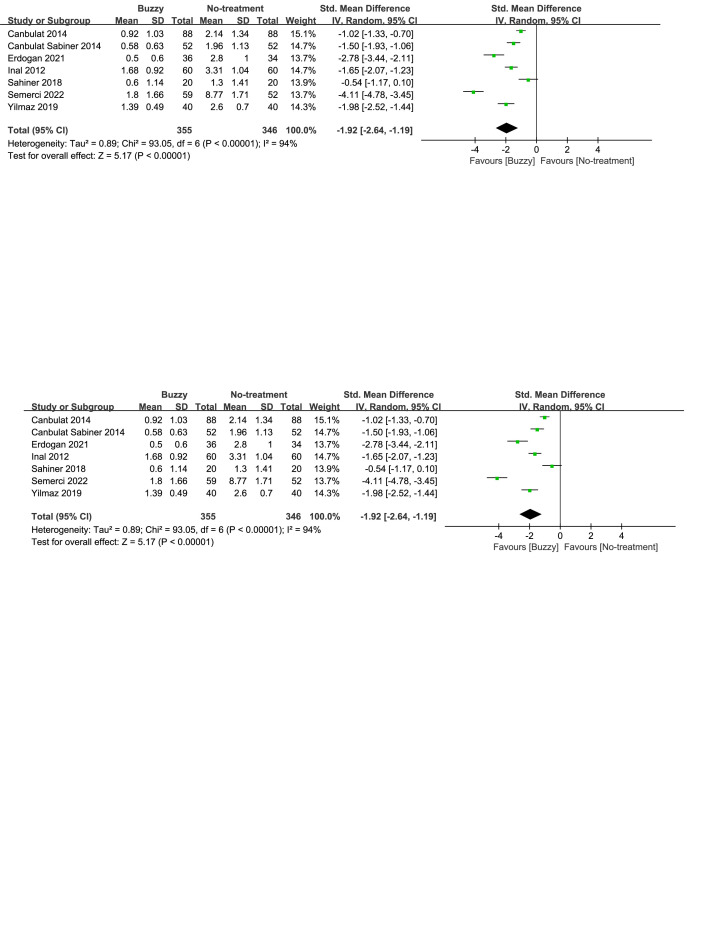


**Figure:**
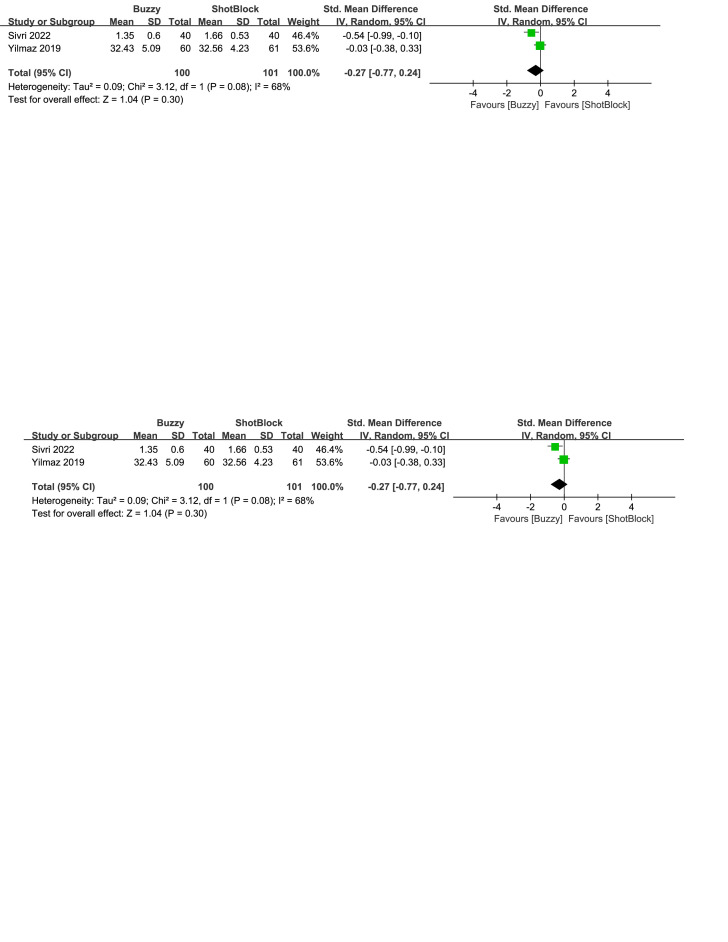


**Figure:**
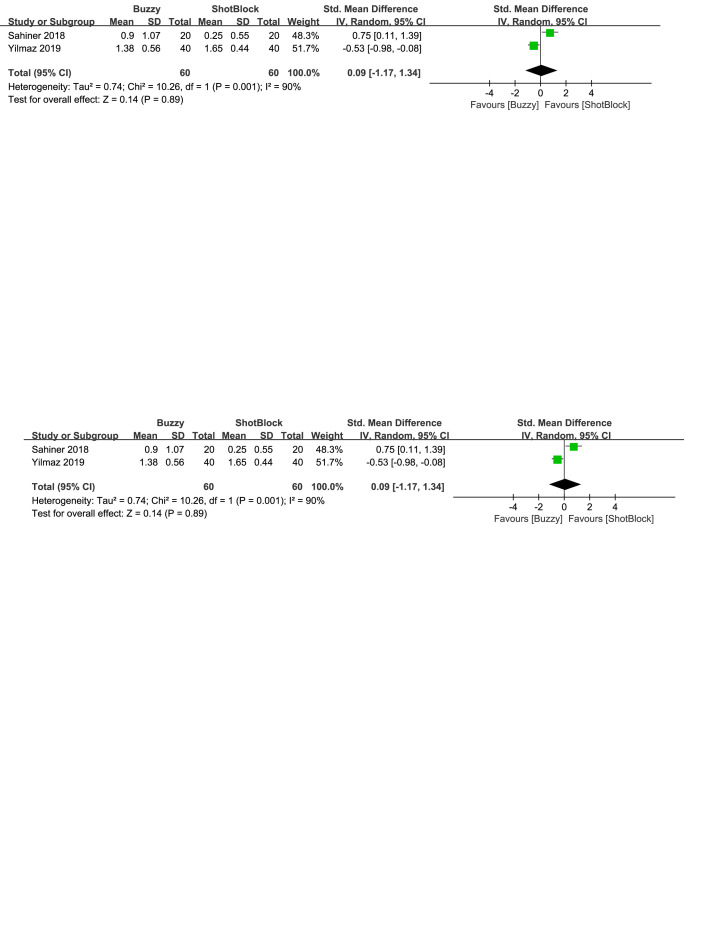


**Figure:**
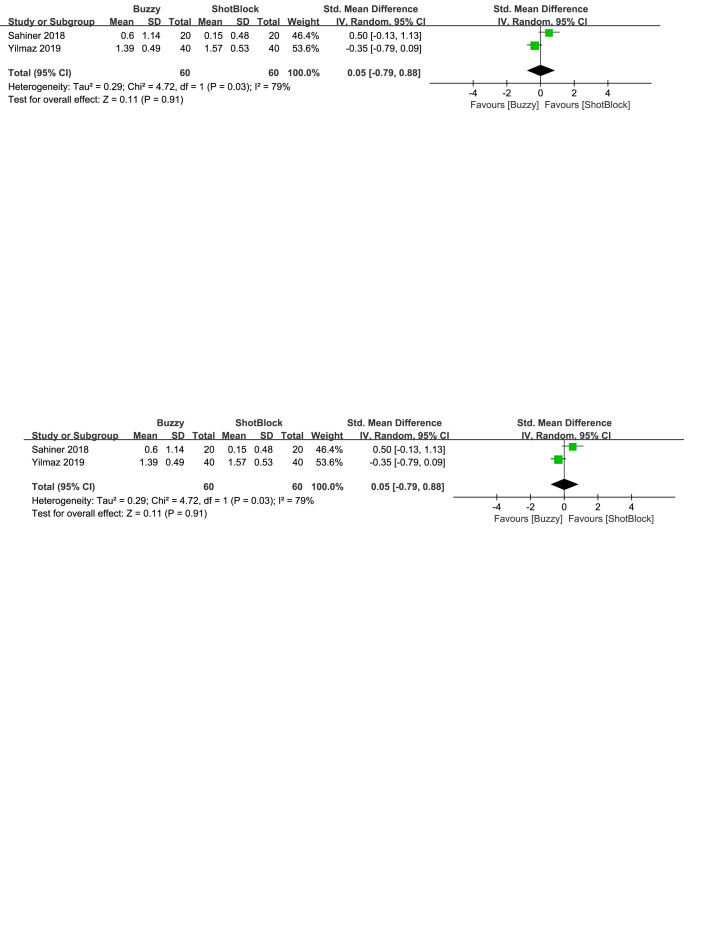


**Figure:**
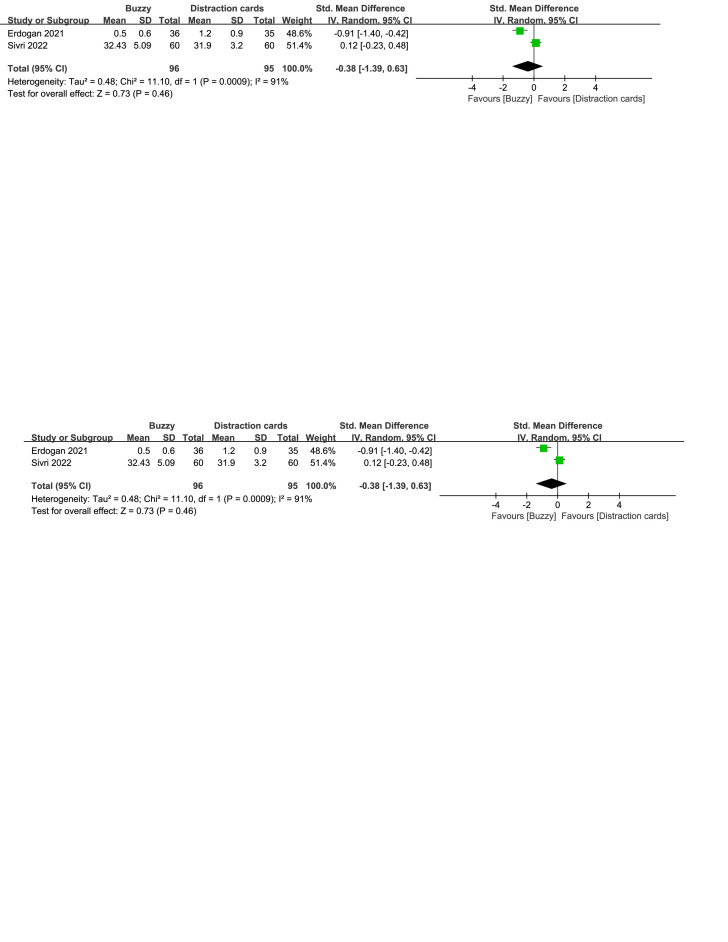


**Figure:**
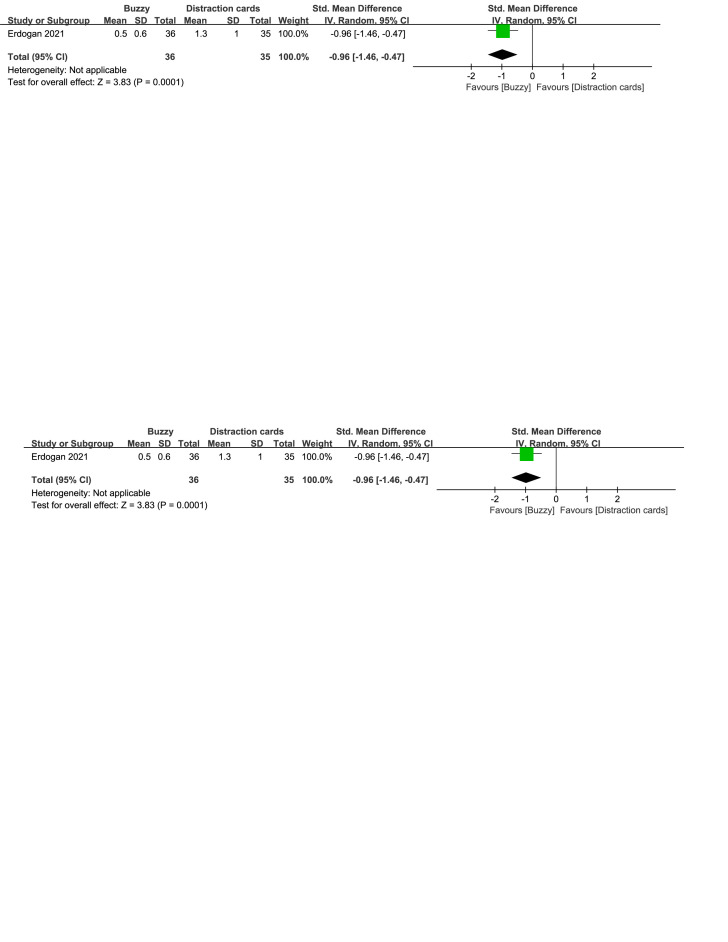


**Figure:**
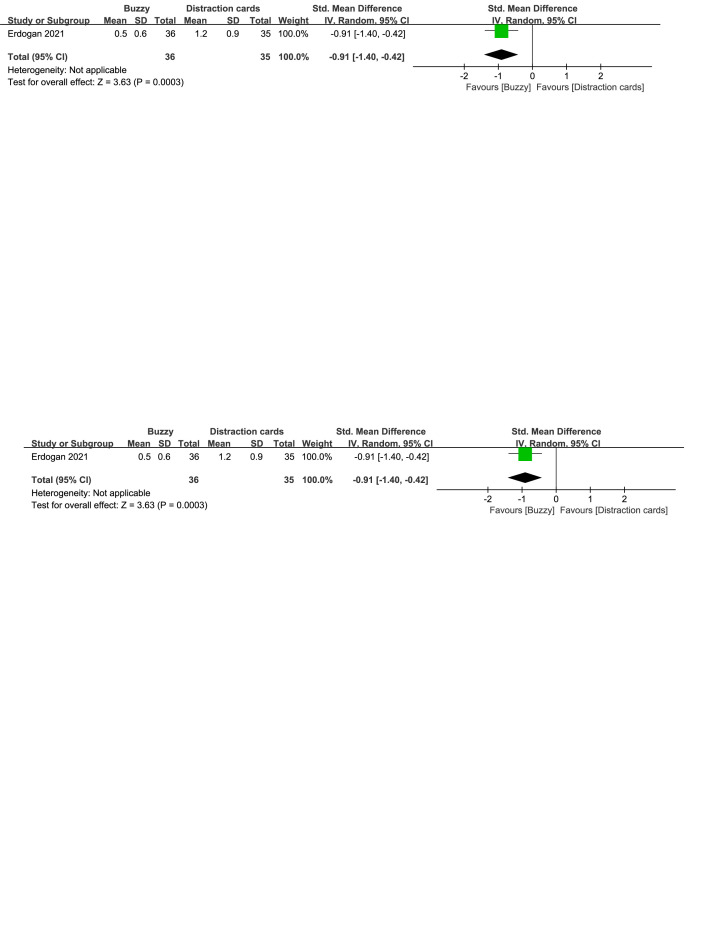


**Figure:**
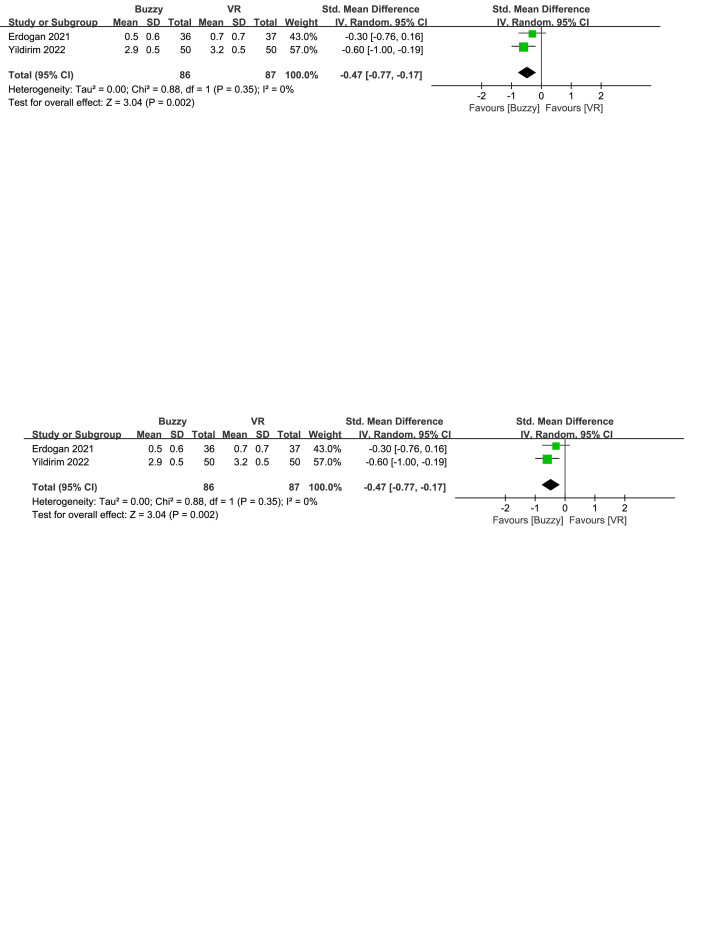


**Figure:**
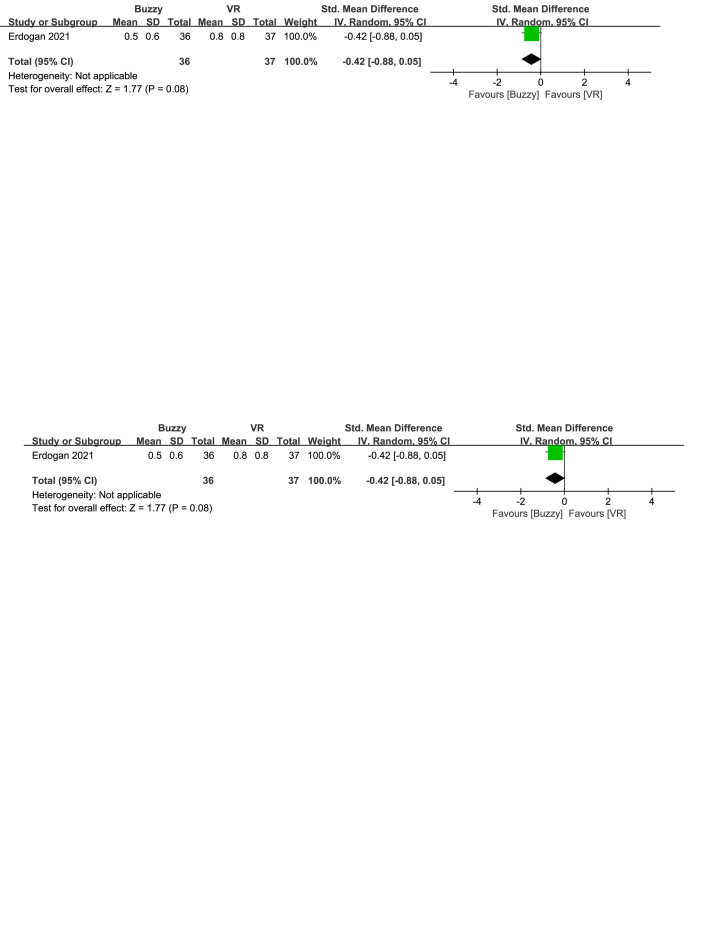


**Figure:**
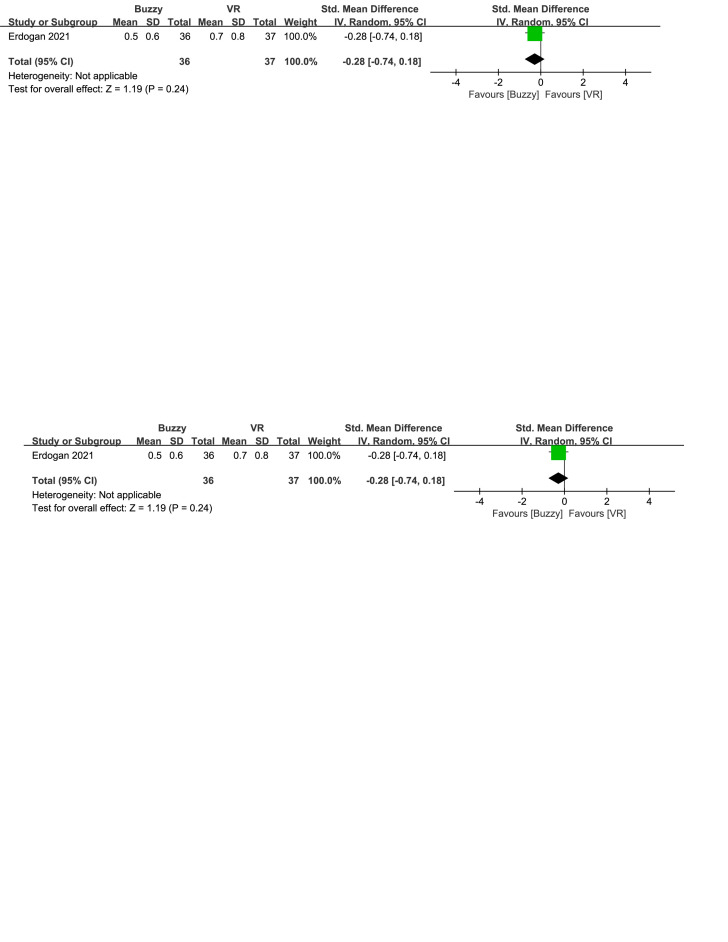


**Figure:**
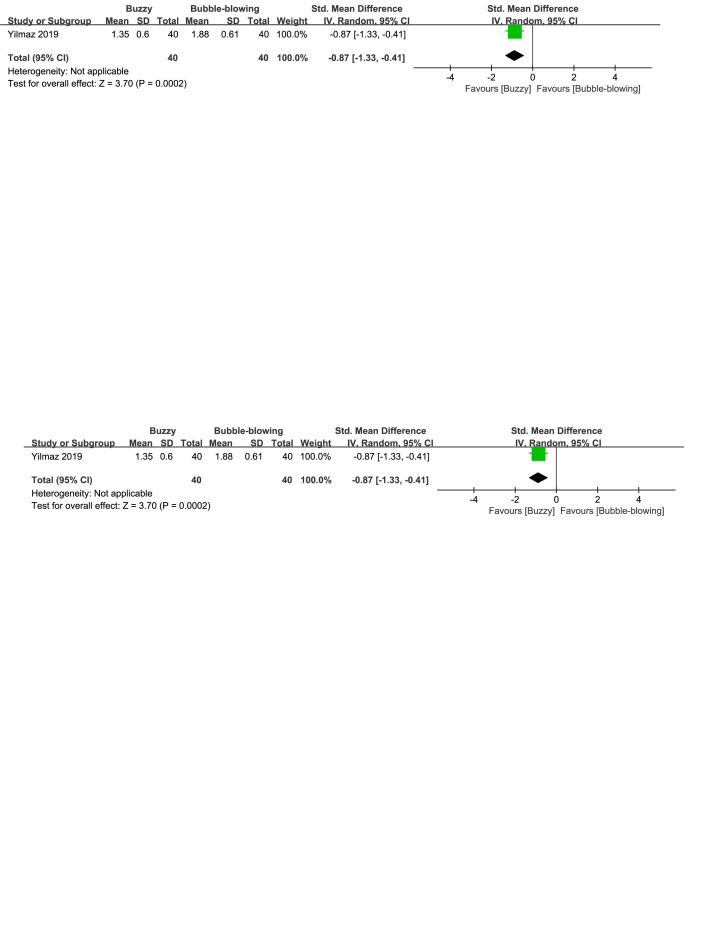


**Figure:**
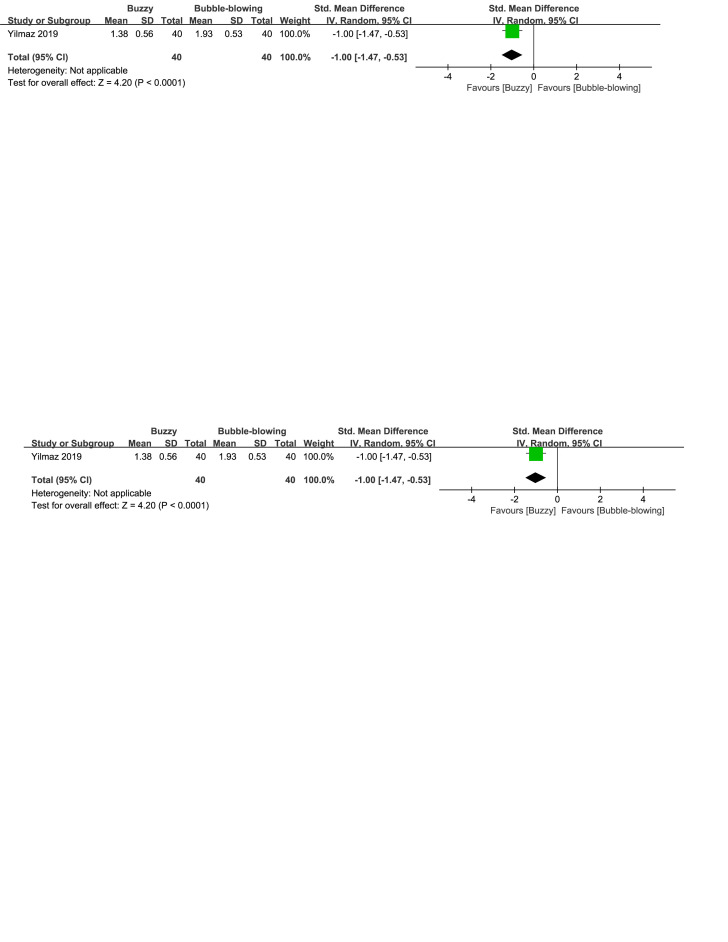


**Figure:**
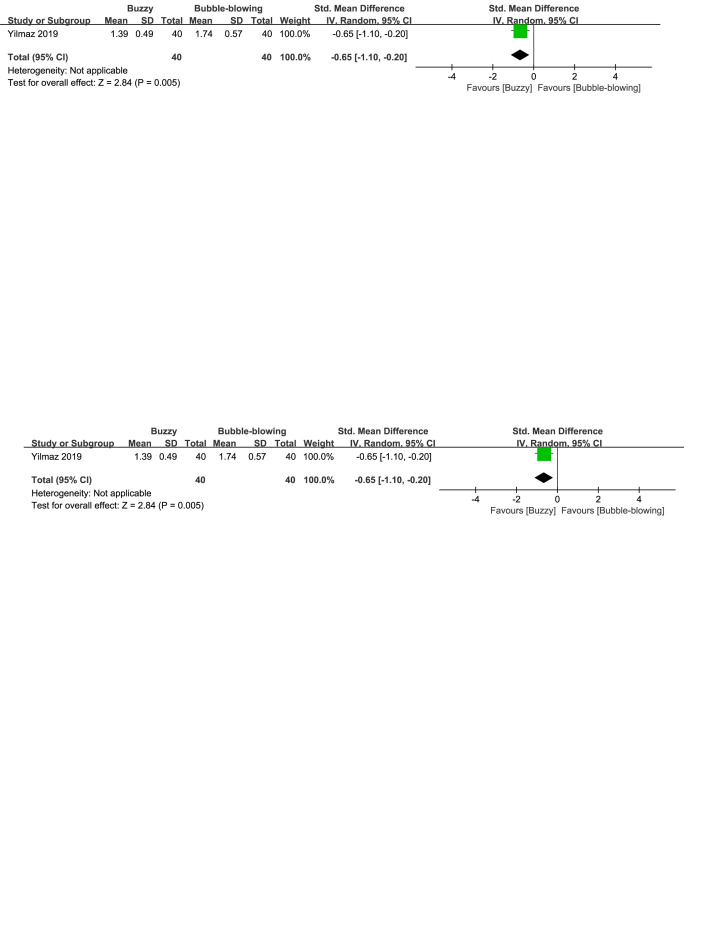


**Figure:**
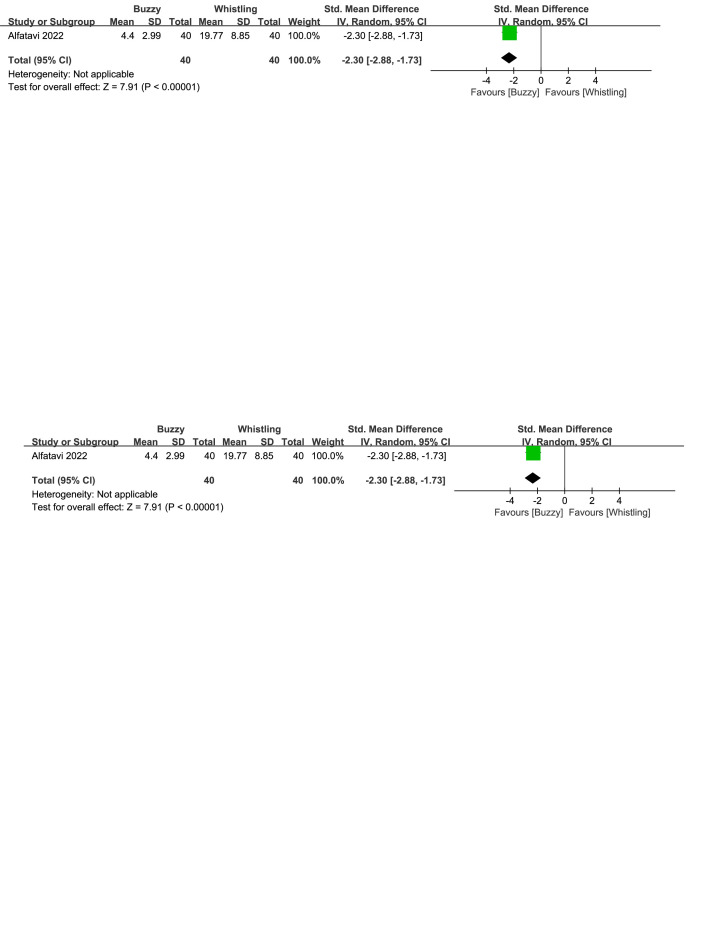


**Figure:**
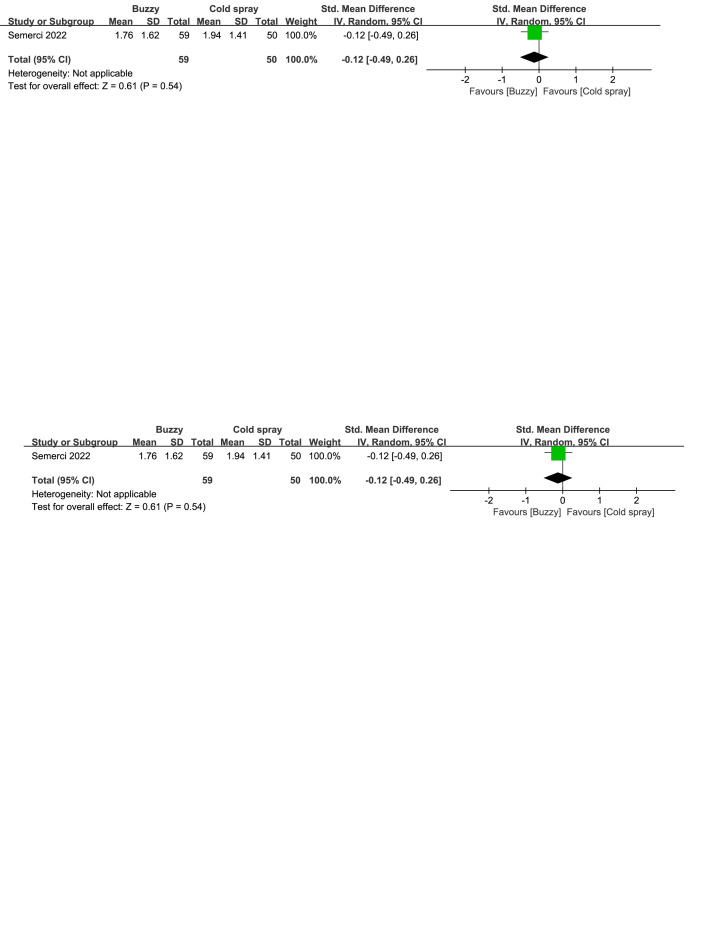


**Figure:**
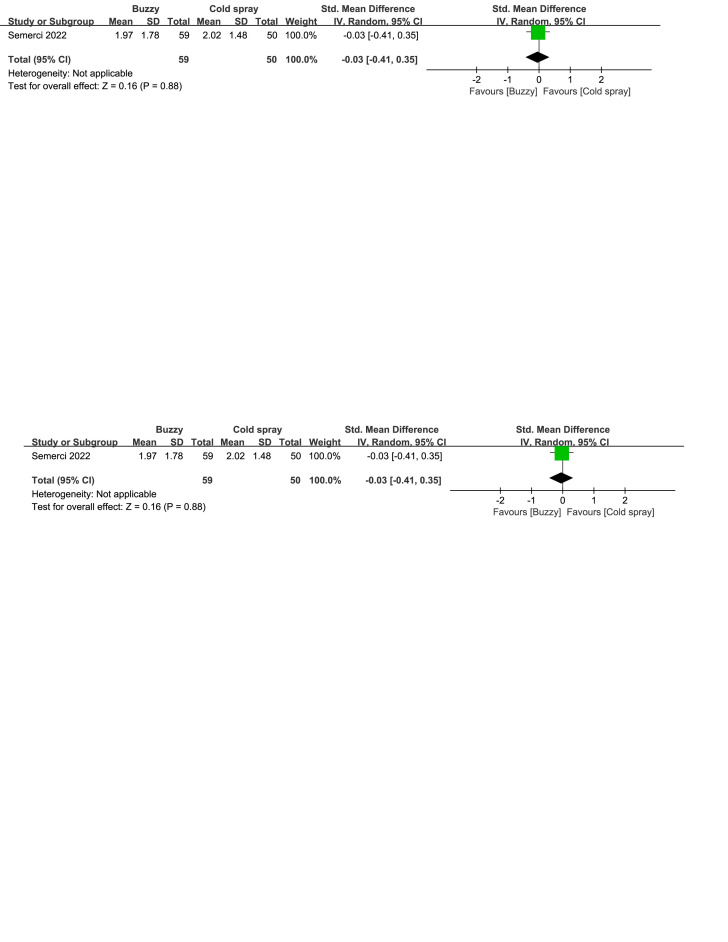


**Figure:**
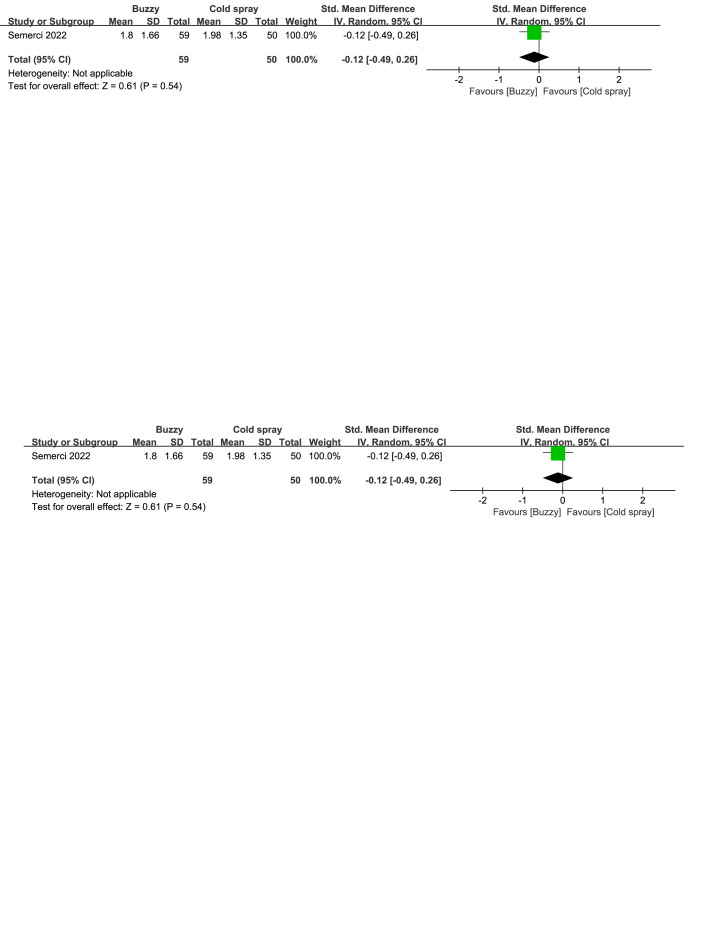


**Figure:**
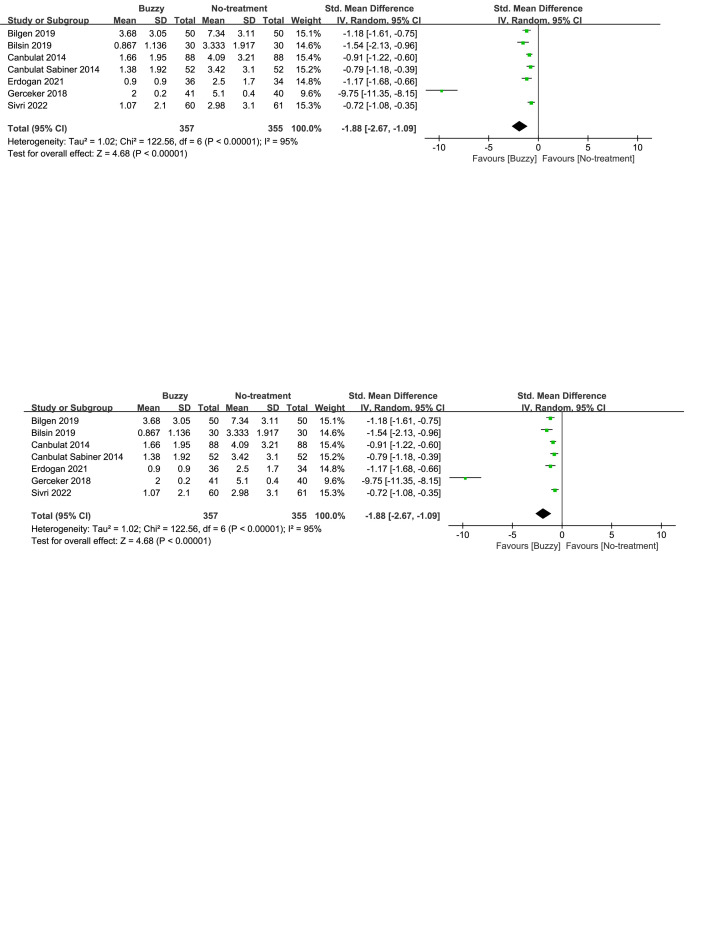


**Figure:**
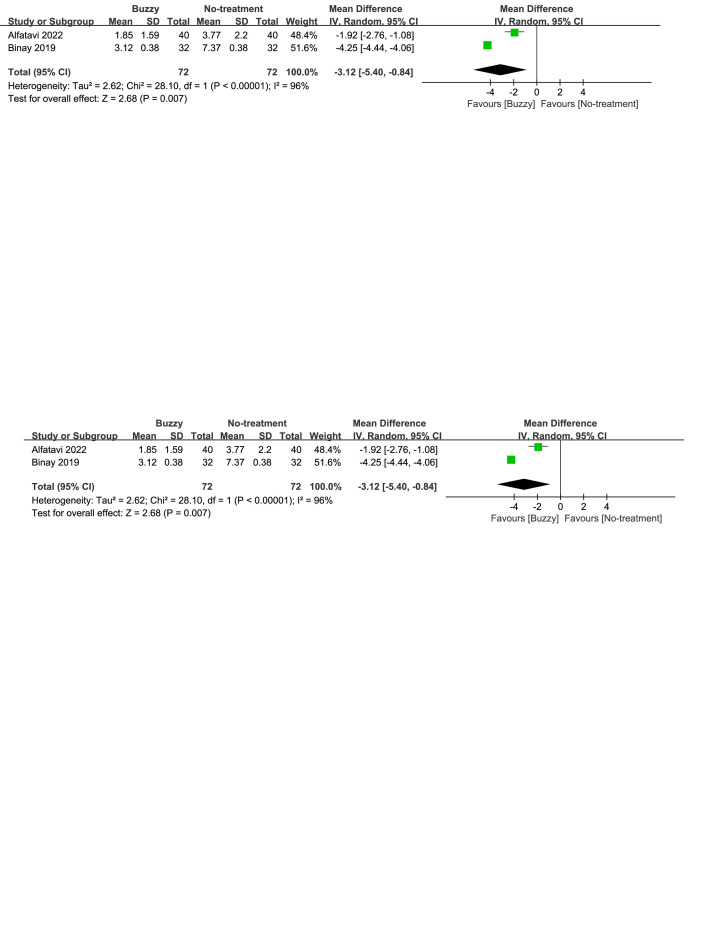


**Figure:**
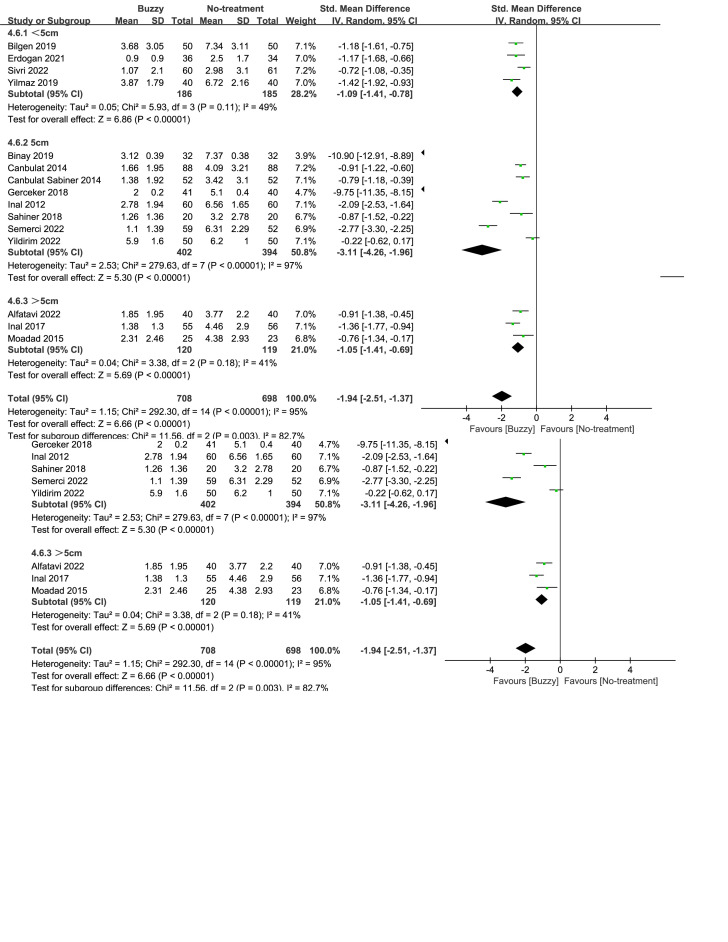


**Figure:**