# Supplementary material for: Effectiveness and safety of Buzzy device in needle-related procedures for children under twelve years of age: A systematic review and meta-analysis
Source: Medicine (Baltimore). 2024 Apr 12;103(15):e37522. doi: 10.1097/MD.0000000000037522 (PMC11018245; doi:10.1097/MD.0000000000037522)
Supplement: Supplementary file 1 [file medi-103-e37522-s043.docx]

**Search strategy**

Title: Effectiveness and Safety of Buzzy® Device Application for Needle-Related Procedures in Children Under 12 Years Old: A Systematic Review and Meta-Analysis

| **Database** | **Search strategy** | **Number of studies included** |
| --- | --- | --- |
| PubMed | ((Buzzy) OR ((((Vibration) AND ((Cold Temperature) OR ((((Cold Temperatures) OR (Temperature, Cold)) OR (Temperatures, Cold)) OR (Cold)))) OR ((Vibration) AND ((((((Cryotherapies) OR (Cold Therapy)) OR (Cold Therapies)) OR (Therapies, Cold)) OR (Therapy, Cold)) OR (Cryotherapy)))) OR ((Vibration) AND ((Refrigeration) OR ((((Cold Chain) OR (Chain, Cold)) OR (Chains, Cold)) OR (Cold Chains)))))) AND (((Infant) OR (Children)) OR ((((((((((((((((Adolescents) OR (Adolescence)) OR (Teens)) OR (Teen)) OR (Teenagers)) OR (Teenager)) OR (Youth)) OR (Youths)) OR (Adolescents, Female)) OR (Adolescent, Female)) OR (Female Adolescent)) OR (Female Adolescents)) OR (Adolescents, Male)) OR (Adolescent, Male)) OR (Male Adolescent)) OR (Male Adolescents))) | 203 |
| Web of Science | ((TS=(((Cryotherapy OR cryotherapie OR “Cold Therapy” OR “Cold Chain” OR Refrigeration OR Cold) AND Vibration) OR Buzzy) AND (Adolescent OR Adolescence OR Teen OR Teenager OR Youth OR “Adolescents, Female” OR “Adolescent, Male”) | 144 |
| Embase | ('infant'/exp OR infant OR 'infants'/exp OR infants OR 'child'/exp OR child OR 'children'/exp OR children OR 'adolescent'/exp OR adolescent OR 'adolescents'/exp OR adolescents OR 'adolescence'/exp OR adolescence OR teens OR teen OR teenagers OR 'teenager'/exp OR teenager OR 'youth'/exp OR youth OR youths) AND (buzzy OR (('vibration'/exp OR vibration) AND ('cold'/exp OR cold OR 'cryotherapy'/exp OR cryotherapy OR 'refrigeration'/exp OR refrigeration))) | 225 |
